# Detection of MSI signals from peripheral blood for monitoring response to immune checkpoint blockade therapy in patients with advanced microsatellite‐unstable gastrointestinal cancers: A pilot study

**DOI:** 10.1002/ijc.70387

**Published:** 2026-02-16

**Authors:** Aysel Ahadova, Lena Bohaumilitzky, Thomas Walle, Joscha A. Kraske, Mirjam Tariverdian, Ulrike Ganserer‐Schmitt, Ingrid Hausser‐Siller, Vera Fuchs, Nina Nelius, Gizem Mehtap Erisen, Johannes Gebert, Albrecht Stenzinger, Dirk Jäger, Magnus von Knebel Doeberitz, Georg Martin Haag, Elena Busch, Matthias Kloor

**Affiliations:** ^1^ Department of Applied Tumor Biology, Institute of Pathology Heidelberg University Hospital Heidelberg Germany; ^2^ National Center for Tumor Diseases Heidelberg, Department of Medical Oncology Heidelberg University Hospital Heidelberg Germany; ^3^ EMLaboratory, Institute of Pathology Heidelberg University Hospital Heidelberg Germany; ^4^ Institute of Pathology Heidelberg University Hospital Heidelberg Germany; ^5^ German Cancer Consortium (DKTK) and German Cancer Research Center (DKFZ) Heidelberg Germany; ^6^ Clinical Cooperation Unit Applied Tumor Immunity German Cancer Research Center (DKFZ) Heidelberg Germany; ^7^ Department of Hematology, Oncology and Rheumatology Heidelberg University Hospital Heidelberg Germany

**Keywords:** biomarkers, extracellular vesicles, gastrointestinal cancers, immune checkpoint blockade, microsatellite instability

## Abstract

Microsatellite instability (MSI) is associated with high immunogenicity in tumors due to the abundance of neoantigens, making MSI cancers particularly responsive to immune checkpoint blockade (ICB) therapy. However, a substantial proportion of patients with MSI gastrointestinal (GI) adenocarcinomas do not benefit from ICB, and non‐invasive biomarkers for monitoring treatment response are lacking. This study investigated the utility of MSI detection in extracellular vesicle (EV) DNA as a liquid biopsy‐based approach for therapy monitoring in patients with advanced MSI GI cancers undergoing ICB therapy and compared it with the analysis of cell‐free (cf) DNA. Blood (*n* = 52) and tumor samples (*n* = 16) were collected from 19 patients before and during therapy. Plasma from 18 patients and 30 healthy controls was analyzed for MSI using four diagnostic mononucleotide markers in both EV DNA and cell‐free DNA (cfDNA). MSI was detected in EV DNA in 8 out of 18 patients and in cfDNA in 9 out of 18 patients, with high concordance (93%) between the two approaches. None of the healthy controls showed MSI signals. MSI detection was more frequent before therapy initiation compared to during therapy time points, and a transition from MSI to non‐MSI status during treatment was associated with clinical benefit or objective response. This switch often occurred before the first staging at 3 months. Thus, MSI analysis in EV DNA is a promising minimally invasive tool for real‐time monitoring of ICB therapy response in MSI GI cancer patients, yielding results comparable to cfDNA while providing handling advantages, and warrants further validation in larger cohorts.

## Graphical abstract

What's new?

A substantial proportion of patients with microsatellite‐unstable gastrointestinal adenocarcinomas do not benefit from immunotherapy. This study demonstrates that microsatellite instability can be reliably detected in plasma‐derived extracellular vesicle DNA from patients with microsatellite‐unstable gastrointestinal cancers. Detection of microsatellite instability in both extracellular vesicle and cell‐free DNA dynamically reflected treatment response to immunotherapy, with a shift to non‐microsatellite instability status being an early indicator of clinical benefit. Extracellular vesicle DNA analysis is a promising minimally invasive tool for real‐time monitoring of immunotherapy response in patients with microsatellite‐unstable gastrointestinal cancers, yielding results comparable to those with cell‐free DNA while providing practical advantages.

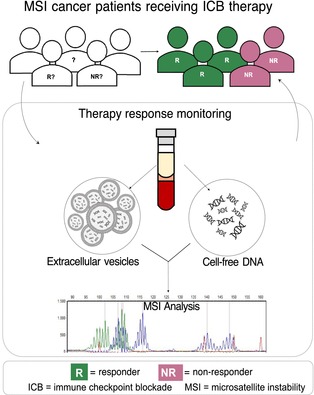

**Abbreviations**
cfDNA: cell‐free DNA
CR: complete response
CRC: colorectal cancer
EVs: extracellular vesicles
GI: gastrointestinal
ICB: immune checkpoint blockade
MMR: mismatch repair
MSI: microsatellite instability
NTA: nanoparticle tracking analysis
OS: overall survival
PD: progressive disease
PFS: progression‐free survival
PR: partial response
SD: stable disease
TEM: transmission electron microscopy

## 1 INTRODUCTION

Deficiency of the DNA mismatch repair system (MMR) results in the accumulation of insertion and deletion (indel) mutations at repetitive microsatellite stretches leading to microsatellite instability (MSI). Approximately 15% of all colorectal cancers (CRC) and up to 5% of stage IV gastrointestinal (GI) tumors present with the MSI phenotype.^1^, ^2^, ^3^ MMR deficiency drastically enhances the likelihood of indel mutations at microsatellites in coding regions of the genome and thus triggers the generation of novel frameshift peptides (FSPs).^4^ FSP neoantigens are foreign to the host and can elicit local and systemic T cell responses.^5^, ^6^

High immunogenicity and dense immune infiltration of MSI cancers render patients with such tumors great potential candidates for immune checkpoint blockade (ICB) therapy.^7^ ICB involves disruption of signaling through immune checkpoints, most prominently programmed death (PD)‐1/programmed death ligand‐1, therefore overcoming adaptive immune resistance and promoting anti‐tumor immune response.^8^ However, while some patients respond exceptionally well to PD‐1 inhibition and experience long‐term disease control, other patients do not respond,^9^ and there is a growing need for non‐invasive, dynamic biomarkers that can accurately reflect treatment response and emerging resistance.^10^, ^11^, ^12^

Approaches based on characterization of cell‐free DNA (cfDNA) have recently become a matter of extensive research.^13^, ^14^, ^15^ In particular, several studies analyzing the utility of cfDNA‐based MSI detection in patients with solid MSI tumors have been performed, showing that cfDNA‐based MSI analysis is feasible and could be used for ICB response prediction or survival.^16^, ^17^ However, data on the performance of cfDNA‐based MSI detection under ICB therapy are limited.^18^ In addition, several technical and logistic aspects in the isolation of cfDNA may limit its applicability in the clinical scenario.^17^ Extracellular vesicles (EVs) have gained attention as a promising tool for therapy monitoring.^19^ These nanoscale, lipid bilayer‐enclosed particles are released by tumor cells into the circulation and carry molecular cargo reflective of their cellular origin and physiological state.^20^, ^21^, ^22^ As such, EVs offer minimally invasive access to versatile biomarkers with the potential to guide real‐time clinical decisions during ICB therapy.

In this study, we assess the potential of MSI status derived from peripheral blood EVs and cfDNA in monitoring MSI cancer patients' responses to ICB therapy.

## 2 METHODS

### 2.1 Patients

Patients with stage IV GI MSI adenocarcinoma who received treatment with ICB at the National Center for Tumor Diseases (NCT) in Heidelberg, Germany, between January 2016 and October 2021 were included in this analysis. Blood samples were obtained from 19 patients and 30 healthy control individuals.

### 2.2 Isolation and analysis of extracellular vesicles

Ethylenediaminetetraacetic acid (EDTA) blood samples (9 mL tubes) were prepared for EV isolation by centrifugation (2500 × *g* and 10,000 × *g*, each for 10 min at 4°C). Total Exosome Isolation reagent (Thermo Fisher Scientific, USA) was added to 1 mL of the cleared plasma supernatant according to the manufacturer's instructions. Precipitated EVs were obtained by centrifugation (20,000 × *g*, 1 h at 4°C) and the resulting pellet was resuspended in PBS for downstream analyses. A subset of EV samples (*n* = 2; P9 and P22) underwent treatment with 0.15 U/μL DNase I for 30 min at 30°C to remove all surface‐associated DNA. The reaction was stopped by the addition of 0.5 M EDTA and incubation at 70°C for 5 min. Before downstream analysis, EVs were centrifuged at 100,000 × *g* for 70 min and resuspended in PBS.

Isolated EVs were assessed by transmission electron microscopy (TEM) and nanoparticle tracking analysis (NTA). For TEM analysis (negative staining), drops of EVs were left to settle on 200 mesh Formvar coated copper grids, air dried, contrasted with 2% aqueous uranyl acetate (Merck KGaA, Germany) and examined at 80 kV with a JEM‐1400 transmission microscope (JEOL Ltd., Japan) equipped with a Tietz 2K digital camera (TVIPS, Germany). NTA was performed with the NanoSight LM10 system (Malvern Panalytical GmbH, Germany) equipped with a 405 nm laser and particles were recorded at five different positions for 1 min each.

### 2.3 DNA isolation from EVs

DNA from EVs (patients and healthy controls) was isolated from 48 samples using the QIAamp DNA Kit (Qiagen, Germany). DNA from matching tumor tissue was isolated from manually microdissected tissue using the DNeasy Blood & Tissue Kit (Qiagen, Germany). All kits were used according to the protocol provided by the manufacturer, and DNA concentrations were determined by a spectrophotometer.

### 2.4 Cell‐free (cf) DNA isolation

cfDNA was isolated from 200 μL of plasma of 46 samples with sufficient rest plasma volume using the Maxwell® RSC ccfDNA Plasma Kit according to the manufacturer's instructions. From two samples, plasma volume did not allow for cfDNA extraction. The concentration of cfDNA was determined fluorometrically.

### 2.5 MSI analysis

The MSI status of EVs, cfDNA, and tumors was determined by polymerase chain reaction (PCR)‐based fragment analysis of a mononucleotide marker panel (BAT25, BAT26, and CAT25) as described previously.^23^ Additionally, the BAT40 microsatellite marker was analyzed in a separate reaction. This marker panel was chosen based on its (quasi)monomorphic nature, high sensitivity, and specificity for MSI detection reported in previous literature.^23^, ^24^, ^25^ Analyses were performed on the ABI3130xl genetic analyzer (Applied Biosystems, Germany). DNA isolated from whole blood was used as a normal tissue control.

MSI was scored when novel peaks in more than one of four analyzed markers were observed. MSI analysis of EV and cfDNA was conducted in a blinded manner and information on the individual (patient or healthy control) was only disclosed after the analysis of allelic patterns, and the MSI status was assessed by two independent observers.

### 2.6 Statistics and visualization

Statistical analysis, if not stated otherwise, was performed in GraphPad Prism (version 6.07). All figures, if not stated otherwise, were produced using GraphPad Prism (version 6.07).

## 3 RESULTS

### 3.1 Clinical data

Nineteen patients with stage IV MSI GI adenocarcinoma (16 CRCs, two gastric cancers, one ampullary cancer) who received treatment with ICB were included in this analysis (Table 1). Details on tumor load are listed in Table S1. Median follow‐up was 29 months (range 3–82). Median age in this patient cohort was 67 years (range 45–80 years) with a predominance of female patients (13 women, 6 men). Most patients received pembrolizumab (*n* = 16); three patients received combination therapy with nivolumab and ipilimumab. Eleven patients had received prior chemotherapy for stage IV disease. Disease stabilization was achieved in eight patients (42%), and objective radiographic investigator‐assessed response according to Response Evaluation Criteria in Solid Tumors (RECIST) criteria^28^ was observed in nine patients (47%). Eight patients presented with partial response (42%), and one patient with complete response (5%). Two patients did not benefit from ICB therapy and had progressive disease (11%). For the isolation and analysis of plasma‐derived EVs, 30 healthy control individuals with a median age of 32 years (range 21–55 years) and a predominance of women (23 women, 7 men) were included.

**TABLE 1:** Patient characteristics. 1/2, one/two prior therapy line(s); Bev, bevacizumab; CR, complete response; FIRI, FOLFIRI; FLO, FLO regimen;^26^ FOL, modified FOLFOX regimen;^27^ FOX, FOLFOX; FOXIRI, FOLFOXIRI; ipi/nivo, nivolumab and ipilimumab; OS, overall survival; Pan, panitumumab; PD, progressive disease; pembro, pembrolizumab; PFS, progression‐free survival; PR, partial response; Ram, ramucirumab; SD, stable disease. The cause of death in patients without documented progression was: P1—unknown, P22—cardiogenic shock, P23—cardiogenic shock in septic prosthetic valve endocarditis and P25—septic shock due to cholangiosepsis.

Patient ID	Diagnosis	*BRAF* wt = 0, mut = 1	*KRAS* wt = 0, mut = 1	Metastatic site	Prior therapy lines	ICB	Response at first staging (3 months)	Best response	Objective response yes = 0, no = 1	PFS (months)	OS (months)	Alive at last follow‐up (December 10, 2022) yes = 0, no = 1
P1	CRC	0	1	OTH (local recurrence)	No	pembro	SD	SD	1	13	13	1
P2	CRC	1	0	PER	No	pembro	PD	PD	1	4	29	0
P3	CRC	1	0	PER, ADR, OTH (spleen), LYM	1 (FIRI/Bev)	pembro	PD	PD	1	1	3	n/a (lost to FU)
P4	Gastric cancer	0	0	OSS, OTH (soft tissue)	1 (FLOT)	pembro	PR	PR	0	20	20	0
P5	CRC	0	1	PER, OTH (local recurrence)	No	ipi/nivo	SD	SD	1	18	18	0
P6	Ampullary cancer	0	0	LYM	1 (FOLFIRINOX)	pembro	PR	PR	0	22	22	0
P7	CRC	1	0	LYM	No	pembro	SD	SD	1	23	23	0
P8	CRC	0	1	OTH (local recurrence), LYM, PLE	No	ipi/nivo	SD	SD	1	9	32	0
P9	CRC	0	0	HEP	No	ipi/nivo	PR	PR	0	14	14	0
P19	CRC	0	1	HEP, PUL, PER	2 (FOX, XELOX/Bev)	pembro	PD	PR	0	33	82	0
P20	CRC	0	1	HEP	1 (FOL/Bev)	pembro	PD	PR	0	39	58	1
P21	CRC	0	0	HEP	1 (FOXIRI/Bev)	pembro	PR	CR	0	47	47	0
P22	CRC	0	0	HEP, PER	No	pembro	PR	SD	0	32	32	1
P23	CRC	0	0	PER, LYM	No	pembro	SD	PR	0	32	32	1
P24	CRC	1	0	LYM, OTH (kidney)	1 (FOL/Bev)	pembro	PR	PR	0	65	65	0
P25	CRC	1	0	HEP, LYM, PER	1 (FOL/Bev)	pembro	SD	SD	1	9	9	1
P26	CRC	1	0	PUL, HEP, PER	1 (FIRI/Pan)	pembro	SD	SD	1	20	28	1
P27	Gastric cancer	0	0	LYM, PER	2 (FLO, FIRI/Ram)	pembro	SD	SD	1	15	48	0
P28	CRC	0	0	PUL, HEP, LYM, PER	2 (FIRI/Bev, FOX/Bev)	pembro	PR	PR	0	82	82	0

Abbreviations: ADR, adrenal metastasis; CRC, colorectal cancer; HEP, liver metastasis; ICB, immune checkpoint blockade; LYM, lymph node metastasis; OSS, bone metastasis; OTH, other sites; PER, peritoneal metastasis; PUL, lung metastasis.

### 3.2 Determination and monitoring of patients' MSI status using plasma‐derived EV DNA and cfDNA

**FIGURE 1:**
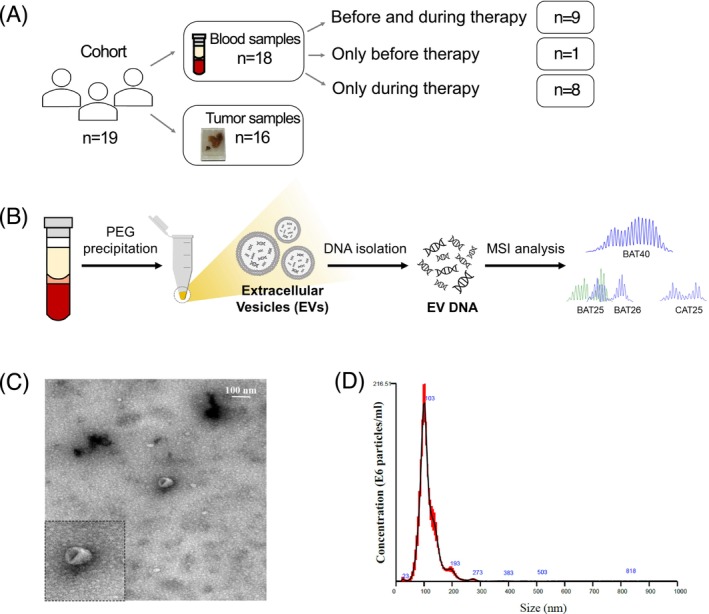
Plasma‐derived extracellular vesicles (EV) preparation and analysis. (A) Schematic overview of the study indicating the number of included patients as well as the number of collected tumor and blood samples. (B) Schematic illustration of the workflow. Detection of microsatellite instability (MSI) in gastrointestinal cancer patients using plasma‐derived EV DNA. (C and D). Analysis of plasma EVs using transmission electron microscopy (C) and nanoparticle tracking analysis (D).

The potential of EVs in therapy response monitoring was assessed using plasma samples from 18 MSI GI cancer patients obtained before and/or during ICB therapy (Figure 1A,B), “before therapy” samples could be obtained from 10/18 patients. A total of 48 plasma samples were collected. Isolated plasma EVs underwent TEM and NTA analyses confirming their cup‐shaped morphology (Figure 1C) and a mean diameter of 114 ± 39 nm (Figure 1D).

Subsequently, EV DNA was isolated and subjected to MSI testing by PCR‐based analysis of allelic profiles of four standard diagnostic mononucleotide markers (BAT40, BAT25, BAT26, and CAT25).^23^, ^24^, ^25^

**FIGURE 2:**
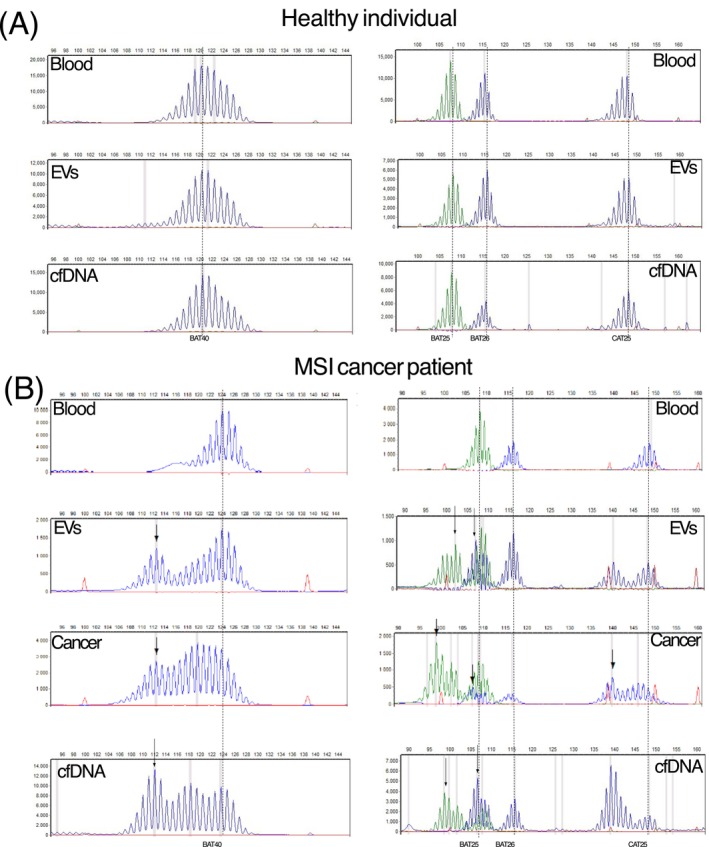
Representative allelic profiles from extracellular vesicles (EV) DNA. (A) EV DNA allelic profiles of a healthy control individual, all showing non‐microsatellite instability (MSI) profiles. (B) Exemplary electropherogram of EV DNA and cell‐free DNA (cfDNA) from an MSI cancer patient showing instability in all four microsatellite markers and coinciding patterns with the respective tumor tissue. The markers BAT25, BAT26, and CAT25 were analyzed in a multiplex PCR, and BAT40 was assessed individually. The amplicon length in base pairs (bp) and fluorescent signal intensity are displayed on the *x* and *y* axis, respectively. Novel peaks indicative of MSI are labeled with arrows.

In parallel, cfDNA isolated from plasma samples, as a widely used method in clinical practice, was also analyzed using the same marker panel (see representative allelic profiles, Figure 2A,B). EV DNA yielded analyzable results in all 48/48 samples, and cfDNA could be examined in 46/48 collected samples (two samples did not have sufficient volume for cfDNA isolation). None of the EVs or cfDNA from 30 healthy control individuals displayed MSI signals. Comparison of MSI analysis between EV DNA and cfDNA showed 93% concordance (43/46 pairs). MSI signals in the EV DNA, indicated by novel peaks in two or more markers compared to the signal in whole blood, were detected in the majority of “before therapy” samples (7/10, 70%), but only in a significantly smaller proportion of “during therapy” samples (6/38, 19%, *p* = .0018, Supporting Information S2: Extended Data 2). Similarly, in the cfDNA, MSI signals were more frequent among samples from “before therapy” time point (6/10, 60%) compared to “during therapy” time points (7/36, 24%, *p* = .0197, Supporting Information S2: Extended Data 2).

**FIGURE 3:**
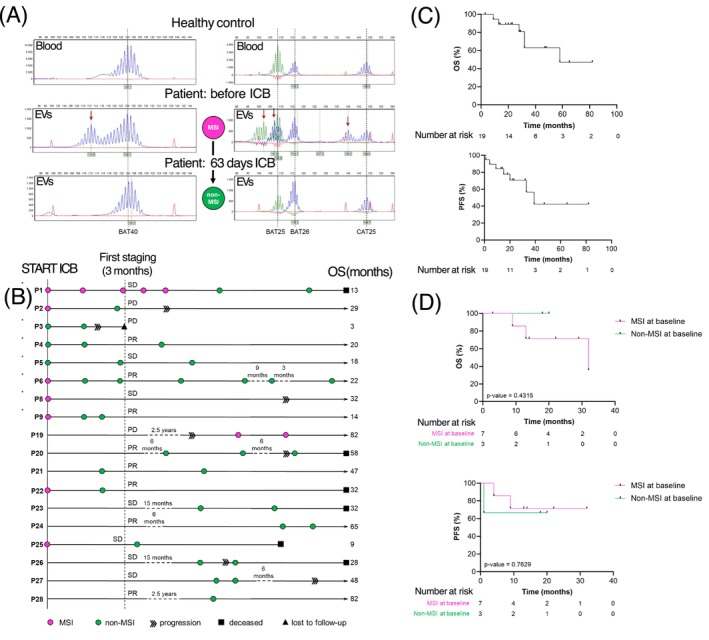
Extracellular vesicles (EV) microsatellite instability (MSI) status and clinical course of MSI cancer patients under immune checkpoint blockade (ICB) therapy. (A) Representative example of changes in the EV MSI status during ICB therapy (P22). After 63 days of treatment no MSI‐associated allelic shifts could be observed in EV DNA. The amplicon length in base pairs (bp) and fluorescent signal intensity are displayed on the *x* and *y* axis, respectively. Novel peaks indicative of MSI are labeled with red arrows. (B) The MSI status was assessed using EV DNA at different time points before and during ICB. (C) Overall (OS) and progression‐free (PFS) survival curves of MSI GI cancer patients receiving ICB therapy. (D) OS and PFS curves of patients with available plasma sample for EV‐based analysis at baseline depending on the loss of EV MSI signals under ICB therapy. No significant difference in OS (log‐rank test, *p* = .1700) and PFS (log‐rank test, *p* = .4624) between the two patient groups were found. PR, partial response; SD, stable disease.

Importantly, four samples with positive MSI signals during therapy originated from the same patient (P1), whose samples eventually switched to non‐MSI status after 200 days of therapy, and the patient showed stable disease (SD) as best response over the entire period of follow‐up. A loss of EV MSI signals upon continued ICB treatment (representative analysis: Figure 3A) could be observed in 6/7 patients with MSI status of the “before therapy” sample, while in 1/7 patients no sample could be obtained during therapy. The observed switch was found to be predominantly correlated with an objective therapy response (P6, P9, and P22) or SD (P1 and P25) (Figure 3B). In four patients (P2, P6, P9, and P22), the switch from MSI to non‐MSI in EV DNA occurred before the first staging at 3 months (Figure 3B).

Additionally, matching tumor tissue samples for MSI analysis were available from 16/18 patients. Importantly, the allelic shifts in the microsatellite marker panel displayed high concordance between tumor tissue DNA and the corresponding EV and cfDNA, strongly supporting the vesicles' and cfDNA's tumor origin (Figure 2B, Supporting Information S1 and S3: Extended Data 1 and 3). The detection of MSI signals in isolated EVs treated with DNase I to remove DNA attached to the EV surface demonstrated that MSI DNA is present in the lumen of tumor‐derived EVs (Figure S1).

### 3.3 Survival analysis in patients receiving ICB therapy

Included MSI cancer patients presented with a median overall survival (OS) after initiation of ICB of 29 months (range 3–82). Median progression‐free survival (PFS) was 20 months (range 1–82 months) with some patients still benefiting at data cut‐off (Figure 3C). In the subgroup analysis, no significant difference in OS or PFS was observed between patients with and without a loss of EV MSI signals during therapy (Figure 3D).

**FIGURE 4:**
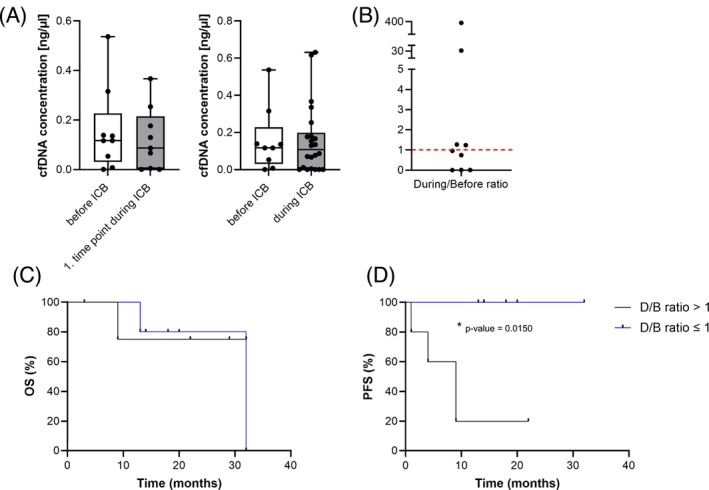
Cell‐free DNA (cfDNA) concentrations and their association with survival. (A) cfDNA concentrations in samples before and during immune checkpoint blockade (ICB) therapy. (B) During/Before ICB (*D*/*B*) ratio of each patient's cfDNA samples. (C) Overall Survival of patients with a *D*/*B* ratio >1 compared to patients with a ratio ≤1. (D) Progression‐free survival (PFS) of patients with a *D*/*B* ratio >1 compared to patients with a ratio ≤1. Patients with a *D*/*B* ratio ≤1 showed a significantly better PFS (*p* = .0150).

Independent from the presence of MSI signals, we also measured the cfDNA concentration in samples obtained before and during therapy (Figure 4A,B). Interestingly, although the OS did not differ in patients depending on the cfDNA during therapy to before therapy (*D*/*B*) ratio (Figure 4C), patients presenting with a *D*/*B* ratio of ≤1 showed a better PFS compared to patients with *D*/*B* ratio >1 (Figure 4D).

## 4 DISCUSSION

Our study demonstrates that MSI can be detected in plasma‐derived EVs from MSI cancer patients. Isolated plasma EVs presented with typical morphology and the expected size.^29^ EV‐based MSI detection was particularly sensitive before ICB therapy start, as most of the analyzed samples (7/10) displayed MSI signals, whereas during ICB therapy the proportion of EV samples with MSI status was significantly lower (6/38). Although previous studies have already demonstrated MSI‐associated frameshift mutations in EVs derived from CRC cell lines^30^ and somatic mutations (e.g., *KRAS*, *BRAF*, *EGFR*, and *TP53*) in circulating EVs from different bodily fluids,^31^, ^32^, ^33^, ^34^, ^35^ this is the first study that proves the presence of MSI signals in plasma EVs.

Several studies have demonstrated that EV levels or phenotypes may indicate clinical course and ICB treatment response.^36^, ^37^, ^38^, ^39^, ^40^ In our study, seven patients presented with MSI signals in the EV DNA prior to therapy start, and, except for the one patient with missing “during therapy” sample, all patients demonstrated a switch to non‐MSI status during ICB. In four patients loss of MSI signals was detected prior to the first staging at 3 months, which is in line with the study by Cercek et al. reporting rapid responses of rectal cancer patients to PD‐1 blockade within weeks after onset of treatment.^41^ In 5/6 patients with the switch to non‐MSI status objective therapy response or SD could be observed at first staging. The only patient (P2) with a switch but no response (PD as best response) still showed 29 months OS and was alive at study end. A loss of MSI signals may be explained by a decreased number of tumor‐derived plasma EVs carrying MSI DNA due to an ICB‐induced reduction of disease burden. Previous studies have demonstrated that malignant cells release higher amounts of EVs compared to non‐malignant cells, and patients with different types of cancer, for example, ovarian, gastric, and colon cancer, present with elevated numbers of circulating EVs in plasma.^21^, ^42^, ^43^ A high tumor burden prior to therapy response and, thus, high numbers of cancer cell‐derived EVs in circulation presumably facilitates MSI detection. Rapid loss of EV‐associated MSI signals might thus indicate the potential of EVs for predicting therapy response from peripheral blood shortly after therapy initiation.

Notably, previous studies have demonstrated sensitive detection of MSI in cfDNA from plasma applying next‐generation sequencing.^44^, ^45^ However, studies systematically assessing both cfDNA and EV‐based methods and comparing their performance in patients receiving ICB therapy have been lacking. In our study, we could demonstrate high concordance between EV‐based and cfDNA‐based MSI detection. While both methods yielded robust results in our study, some practical and biological aspects might favor the utilization of EV DNA in clinical practice. In contrast to short‐lived cfDNA (estimated half‐life <2 h) demanding fast and specialized sample collection, EV DNA is protected by the lipid bilayer and, thus, is more stable.^46^, ^47^ Further, EVs are released from metabolically active cells and they may be a more representative source of tumor DNA compared to cfDNA which is released by dying cells.^46^ Lastly, the rich molecular cargo of EVs may harbor potential to streamline various analyses beyond or complementary to DNA or MSI analysis. Of note, integration of EVs in the clinical routine would not change the established patient‐facing clinical flow as such pre‐therapy sampling is usually part of the common clinical practice. On the healthcare provider‐facing end, once isolated, EV‐DNA can be processed with the same downstream workflows used for cfDNA or tissue‐derived DNA (PCR‐based MSI assays), with comparable turnaround times for the analytical step. Taking into account the above mentioned points and the decreasing costs for commercial EV isolation kits, EVs may become highly attractive analytes in the future.

Eight patients did not display EV or cfDNA MSI signals, including three (P3, P4, and P5) that lacked detectable MSI signals prior to treatment. The absence of MSI signals in these individuals may be related to a low tumor load (in P3 and P4) or to the metastatic pattern (peritoneal metastasis in P5). Particularly, a peritoneal metastatic pattern has been previously associated with significantly lower circulating tumor DNA levels possibly attributable to their poor vascularization.^48^, ^49^ On the contrary, one patient (P19) presented with durable MSI signals in the EVs after 3 years on ICB therapy. MSI clones may persist during long‐term PD‐1 blockade likely because even highly immunogenic deficient MMR (dMMR/MSI) tumors contain small subclones or microenvironments with impaired antigen presentation, defective interferon gamma signaling, or local immunosuppression, allowing rare pockets of tumor cells to survive in immune‐controlled dormancy.^50^ As no biopsy samples could be collected during therapy, this possibility could not be analyzed in the current study.

Notably, our microsatellite marker panel consisted of highly sensitive markers, as described previously,^23^, ^24^, ^25^, ^51^, ^52^, ^53^ and strong amplification of microsatellite markers was observed in all patients, indicating that loss of MSI signals was not reflective of the amount of EV‐associated DNA. However, it is conceivable that allele‐specific amplification strategies may enhance the sensitivity of systemic MSI signal detection in the future. No significant influence of prior therapy lines on EV MSI signals was observed.

Our study has limitations. First, the number of time points for blood sample collection and intervals between samplings could not be standardized for all patients. This was unavoidable in our study due to the sequential recruitment upon availability and the clinical routine. Second, the analyzed cohort size was small. However, stage IV MSI cancer represents a rare event in clinical practice, hence, within this disease setting, our analysis of 19 patients is, to our best knowledge, the first comprehensively analyzed cohort of stage IV MSI patients assessed in parallel for EV‐ and cfDNA‐based MSI signals and followed‐up for over 80 months' period. Third, the use of RECIST guidelines as assessment for ICB response may not fully characterize ICB response and long‐term benefits due to the unique mechanism of action based on T cell activation.^54^, ^55^ Improved, immunotherapy‐specific response assessment criteria, which are currently being evaluated in clinical trials, may help to more precisely assess the actual treatment response in ICB patients.

We did not observe any difference in the OS or PFS of patients showing MSI versus non‐MSI EV signals at baseline. Most likely, the mentioned analyses were underpowered and warrant further replication in larger cohorts. Furthermore, the small number of patients per subgroup in the current study precluded a meaningful analysis of the impact of somatic mutations, such as *BRAF* and *KRAS* on the ICB response or EV MSI status switch. Although reports of *KRAS*/*BRAF* mutations impacting the immune infiltration in MSI CRC^56^ support the plausibility of an association of these mutations with ICB response, a recent study did not detect such effects.^57^

In summary, our study demonstrates that MSI signals can be detected in plasma‐derived EVs as well as in cfDNA using mononucleotide‐based fragment length analysis, and changes of MSI status during the course of ICB treatment might be early indicators of therapy response. The performance for dynamic therapy monitoring in patients receiving ICB needs to be validated in multicenter studies.

## AUTHOR CONTRIBUTIONS

**Aysel Ahadova:** Conceptualization; validation; data curation; writing – original draft; writing – review and editing; visualization; supervision; project administration; funding acquisition. **Lena Bohaumilitzky:** Conceptualization; methodology; software; investigation; data curation; writing – original draft; writing – review and editing; visualization; funding acquisition. **Thomas Walle:** Methodology; validation; investigation; resources; writing – review and editing. **Joscha A. Kraske:** Investigation; resources; writing – review and editing. **Mirjam Tariverdian:** Resources; investigation; writing – review and editing. **Ulrike Ganserer‐Schmitt:** Methodology; investigation; resources; writing – review and editing. **Ingrid Hausser‐Siller:** Methodology; resources; writing – review and editing. **Vera Fuchs:** Methodology; validation; investigation; writing – review and editing. **Nina Nelius:** Investigation; methodology; writing – review and editing. **Gizem Mehtap Erisen:** Investigation; methodology; writing – review and editing. **Johannes Gebert:** Methodology; validation; writing – review and editing; supervision. **Albrecht Stenzinger:** Validation; writing – review and editing; supervision. **Dirk Jäger:** Validation; resources; writing – review and editing; supervision. **Magnus von Knebel Doeberitz:** Validation; writing – review and editing; supervision; project administration; funding acquisition. **Georg Martin Haag:** Validation; resources; writing – review and editing; supervision. **Elena Busch:** Conceptualization; validation; resources; data curation; writing – original draft; writing – review and editing; supervision; funding acquisition. **Matthias Kloor:** Conceptualization; validation; data curation; writing – original draft; writing – review and editing; supervision; project administration; funding acquisition.

## FUNDING INFORMATION

This study was performed with grant support from Else‐Kröner‐Fresenius Foundation, Grant number: 2018_A44; German Cancer Aid, Grant number: 70113455; Klaus Tschira Foundation, Grant number: 00.012.2021; Donations against Cancer, NCT Heidelberg and Stiftung für Krebs‐ und Scharlachforschung. The funding bodies had no role in the study design, collection, analysis, or interpretation of the data. Aysel Ahadova is funded by the Advanced Medical Scientist Program of Heidelberg University, Faculty of Medicine. [Correction added on 07 March 2026, after first online publication: The Funding Information has been updated.].

## CONFLICT OF INTEREST STATEMENT

Thomas Walle reports stock ownership for Roche, Bayer, Innate Pharma, Illumina, Fibrogen, 10x Genomics, Astra Zeneca, Merck KGaA, Immatics, Grail; travel funds from Roche, as well as research funding from CanVirex AG, Basel Switzerland and Institut für Klinische Krebsforschung GmbH, Frankfurt, Germany. Albrecht Stenzinger is a member of the advisory board/speaker's bureau of Agilent, Aignostics, Amgen, Astellas, Astra Zeneca, Bayer, BMS, Eli Lilly, Illumina, Incyte, Janssen, MSD, Novartis, Pfizer, Qlucore, QuiP, Roche, Sanofi, Seagen, Servier, Takeda, Thermo Fisher, and reports funding from Bayer, BMS, Chugai, Incyte, and MSD. Georg Martin Haag reports consulting or advisory roles for Bristol‐Myers Squibb, MSD Sharp & Dohme, Lilly, Novartis, Daiichi Sankyo, Servier, and Pierre Fabre, honoraria from Servier, MSD Sharp & Dohme, Lilly, Targos, Bristol‐Myers Squibb, IOMEDICO, MCI Conventions, research funding from DKFZ Heidelberg and MSD Sharp & Dohme, and travel and accommodations from Bristol‐Myers Squibb, Lilly, Servier, MSD Sharp & Dohme, and Daiichi Sankyo, outside the submitted work. The other authors declare no conflicts.

## ETHICS STATEMENT

Informed consent was obtained from all patients, and the study was approved by the local Ethics committee (V5.1S207/2005, S373/2020, S735/2021, S375/2021, and S308/2025).

## Supporting information


**Data S1.** Supporting Information.

**Data S2.** Supporting Information.

**Data S3.** Supporting Information.

**Data S4.** Supporting Information.

## Data Availability

The data generated in this study are available within the article and its Supporting Information [Link], [Link], [Link], [Link]. Further information is available from the corresponding author upon request.
