# Supplementary material for: Detection of MSI signals from peripheral blood for monitoring response to immune checkpoint blockade therapy in patients with advanced microsatellite‐unstable gastrointestinal cancers: A pilot study
Source: Int J Cancer. 2026 Feb 16;158(12):3312–23. doi: 10.1002/ijc.70387 (PMC13106927; doi:10.1002/ijc.70387)
Supplement: Supplementary file 1 — Data S1. Supporting Information. [file IJC-158-3312-s003.pdf]

EXTENDED DATA 1

MSI analysis of EV DNA and cfDNA samples from patients receiving immune checkpoint therapy.

Red arrows indicate shifts in microsatellite peak patterns.

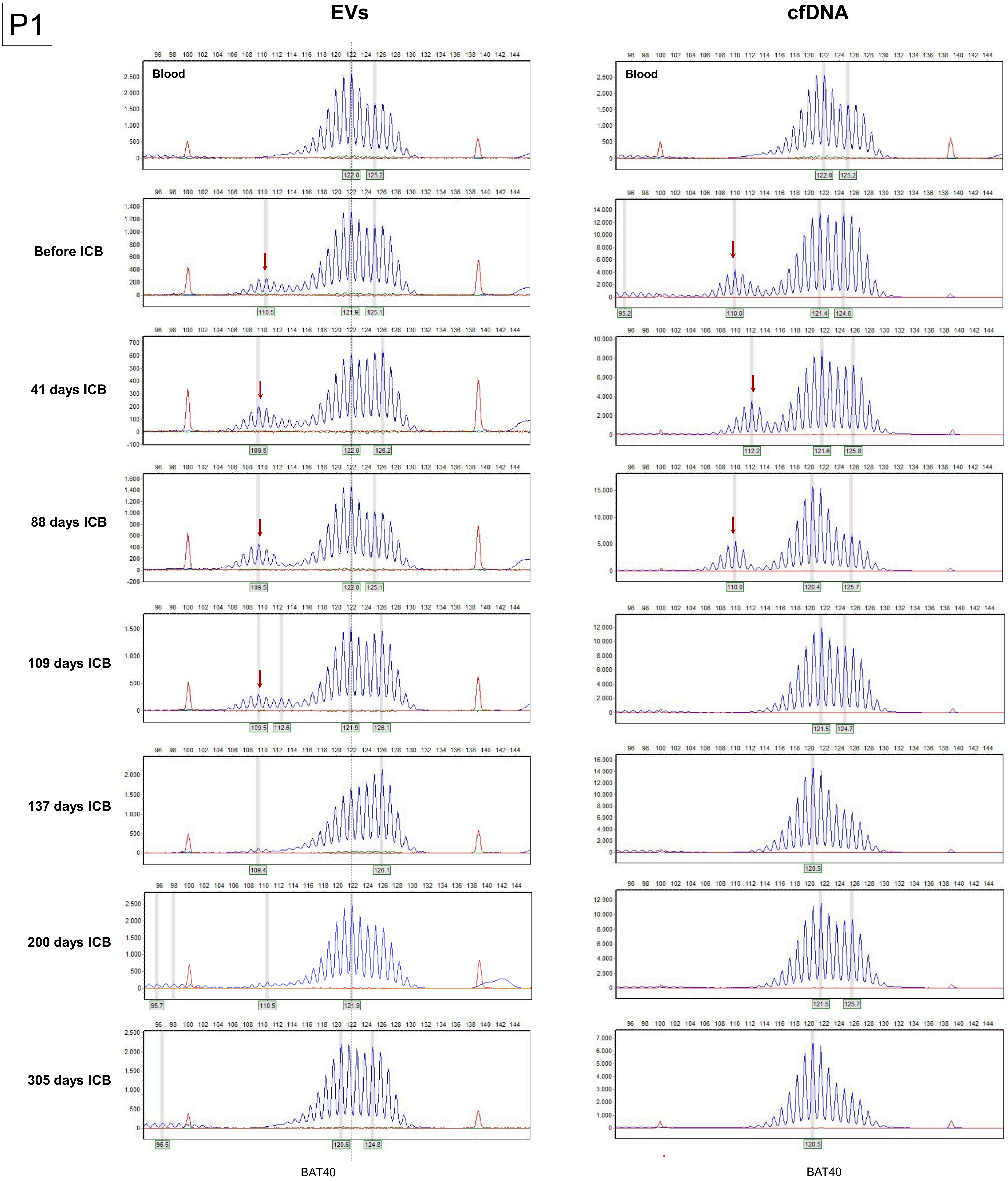
P1
EVs
cfDNA
Blood
Blood
Before ICB
41 days ICB
88 days ICB
109 days ICB
137 days ICB
200 days ICB
305 days ICB
122.0
125.2
122.0
125.2
110.5
121.9
125.1
95.2
110.0
121.4
124.6
109.5
122.0
126.2
112.2
121.6
125.8
109.5
122.0
125.1
110.0
120.4
125.7
109.5
112.6
121.9
126.1
121.5
124.7
109.4
126.1
120.5
95.7
110.5
121.9
121.5
125.7
96.5
120.6
124.8
120.5
BAT40
BAT40

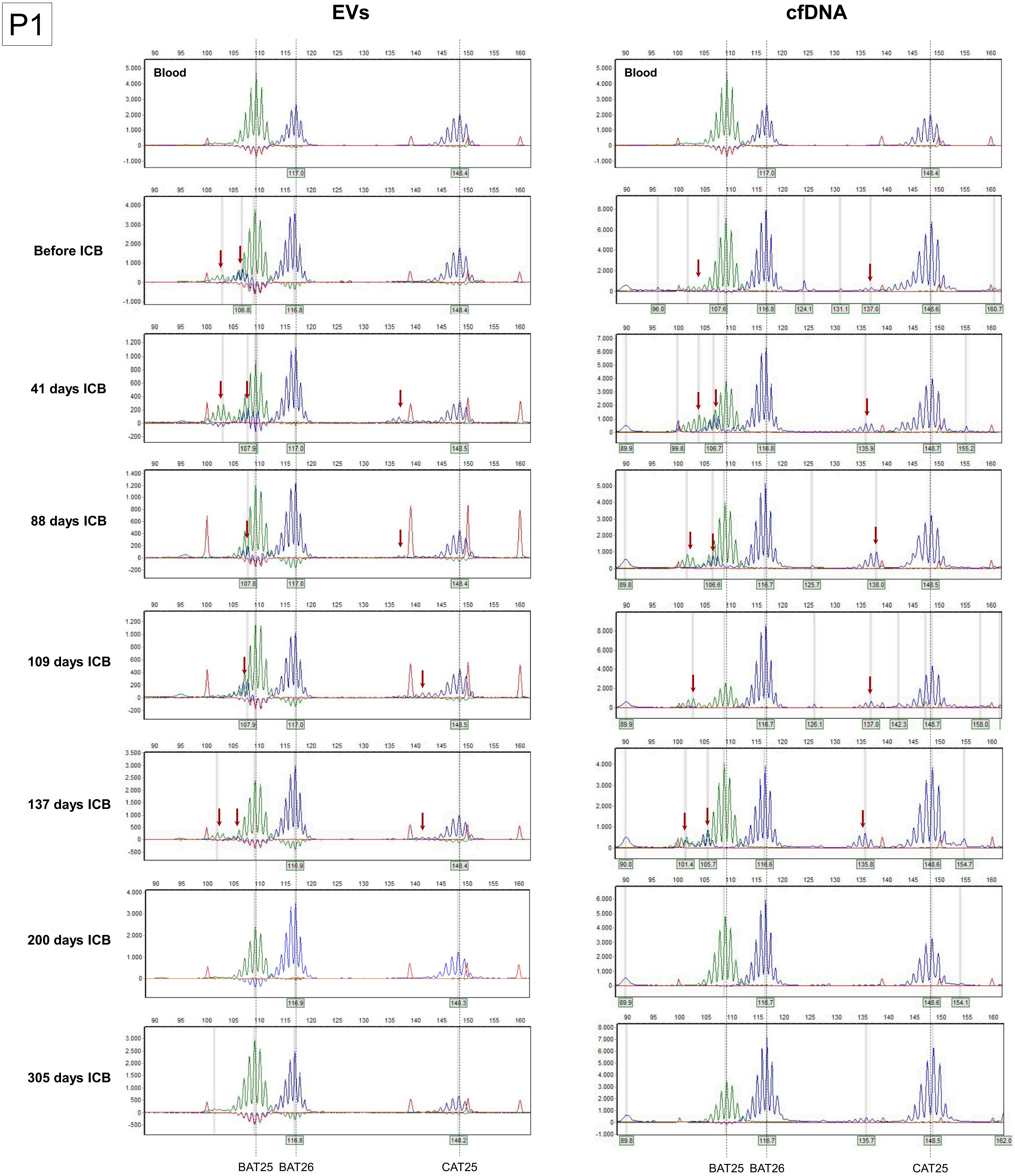
P1
EVs
cfDNA
Blood
Blood
Before ICB
41 days ICB
88 days ICB
109 days ICB
137 days ICB
200 days ICB
305 days ICB
BAT25 BAT26
CAT25
BAT25 BAT26
CAT25

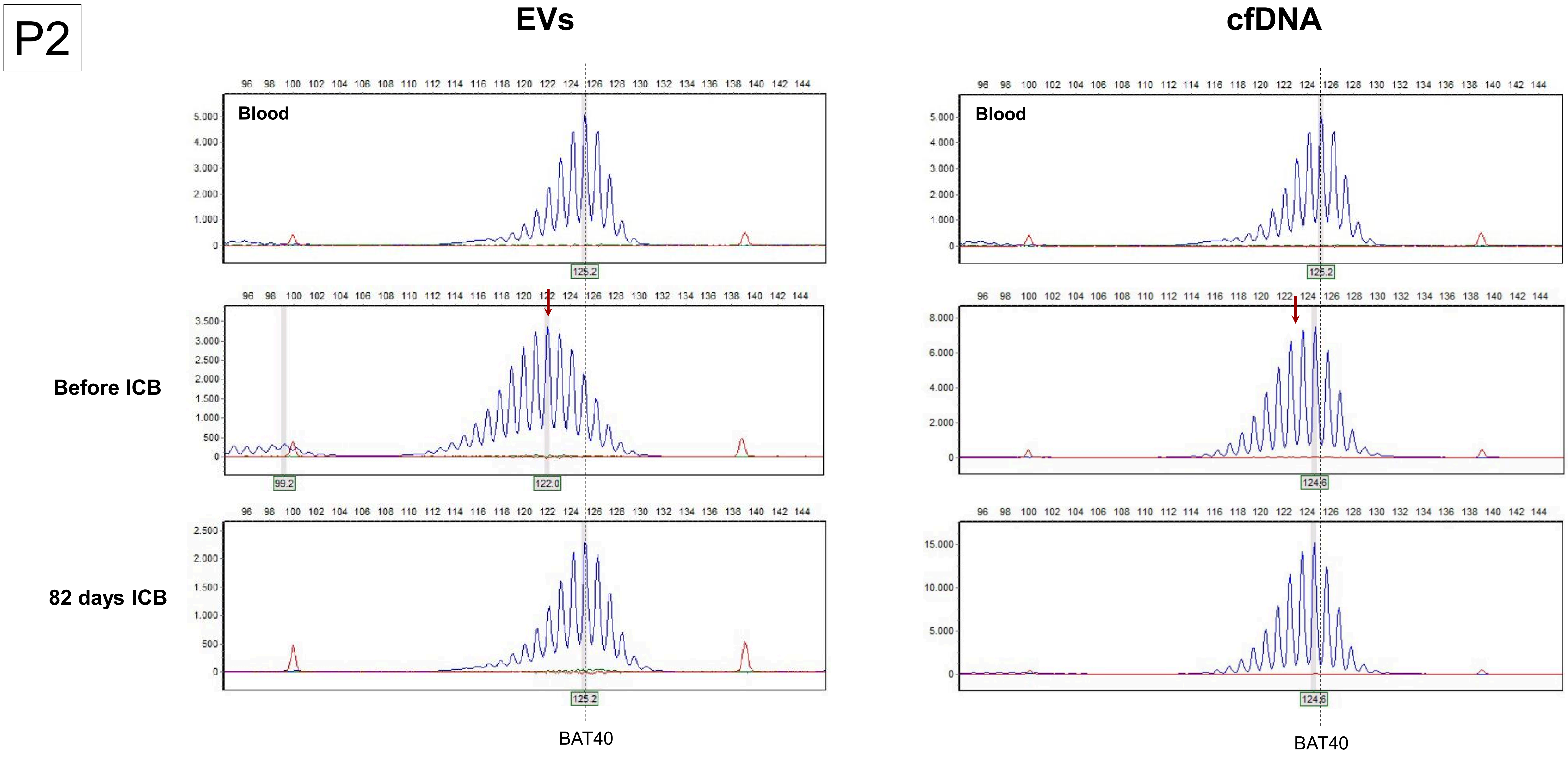
P2
EVs
cfDNA
Blood
Blood
Before ICB
82 days ICB
BAT40
BAT40

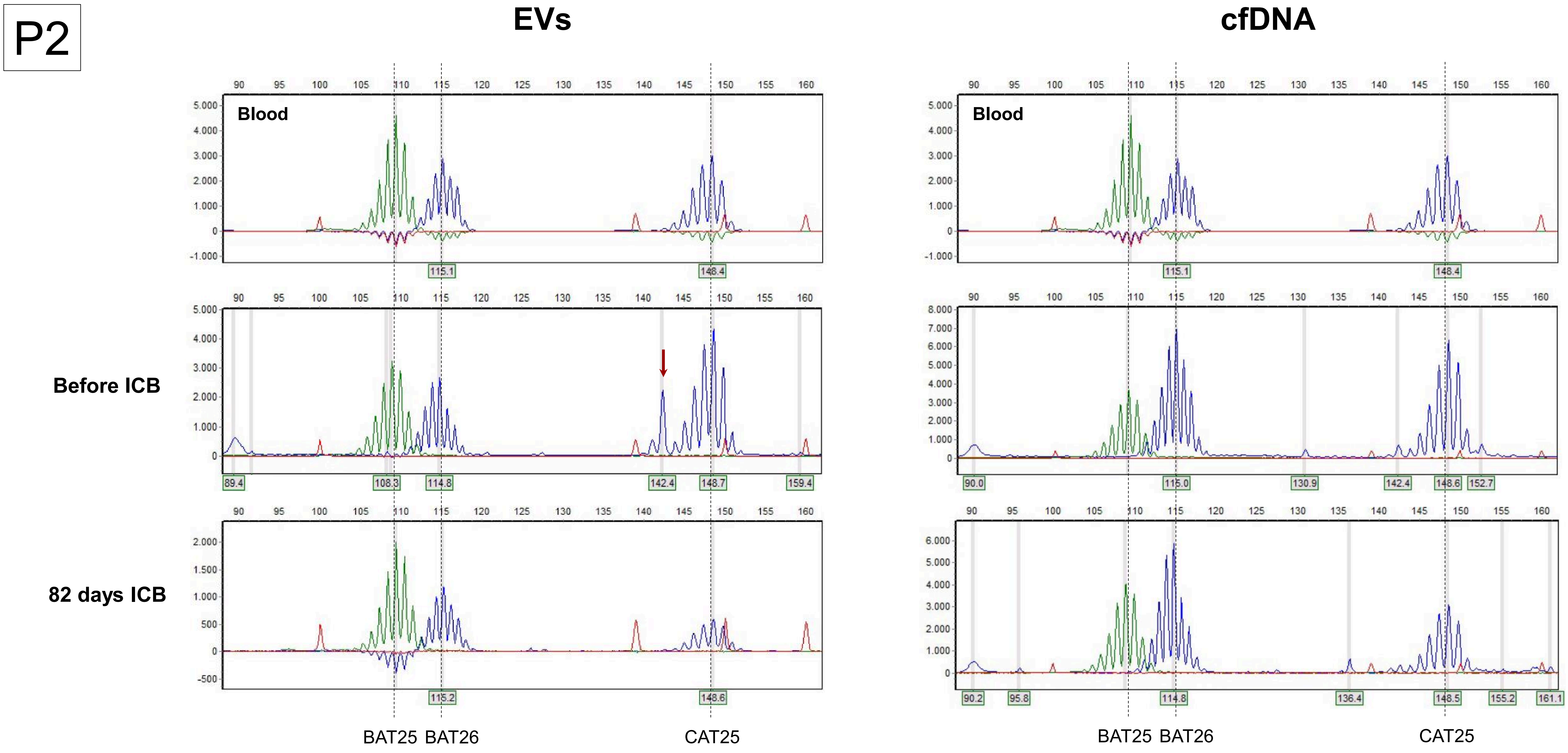
P2
EVs
cfDNA
Blood
Blood
Before ICB
82 days ICB
115.1
148.4
89.4
108.3
114.8
142.4
148.7
159.4
115.2
148.6
115.1
148.4
90.0
115.0
130.9
142.4
148.6
152.7
90.2
95.8
114.8
136.4
148.5
155.2
161.1
BAT25 BAT26
CAT25
BAT25 BAT26
CAT25

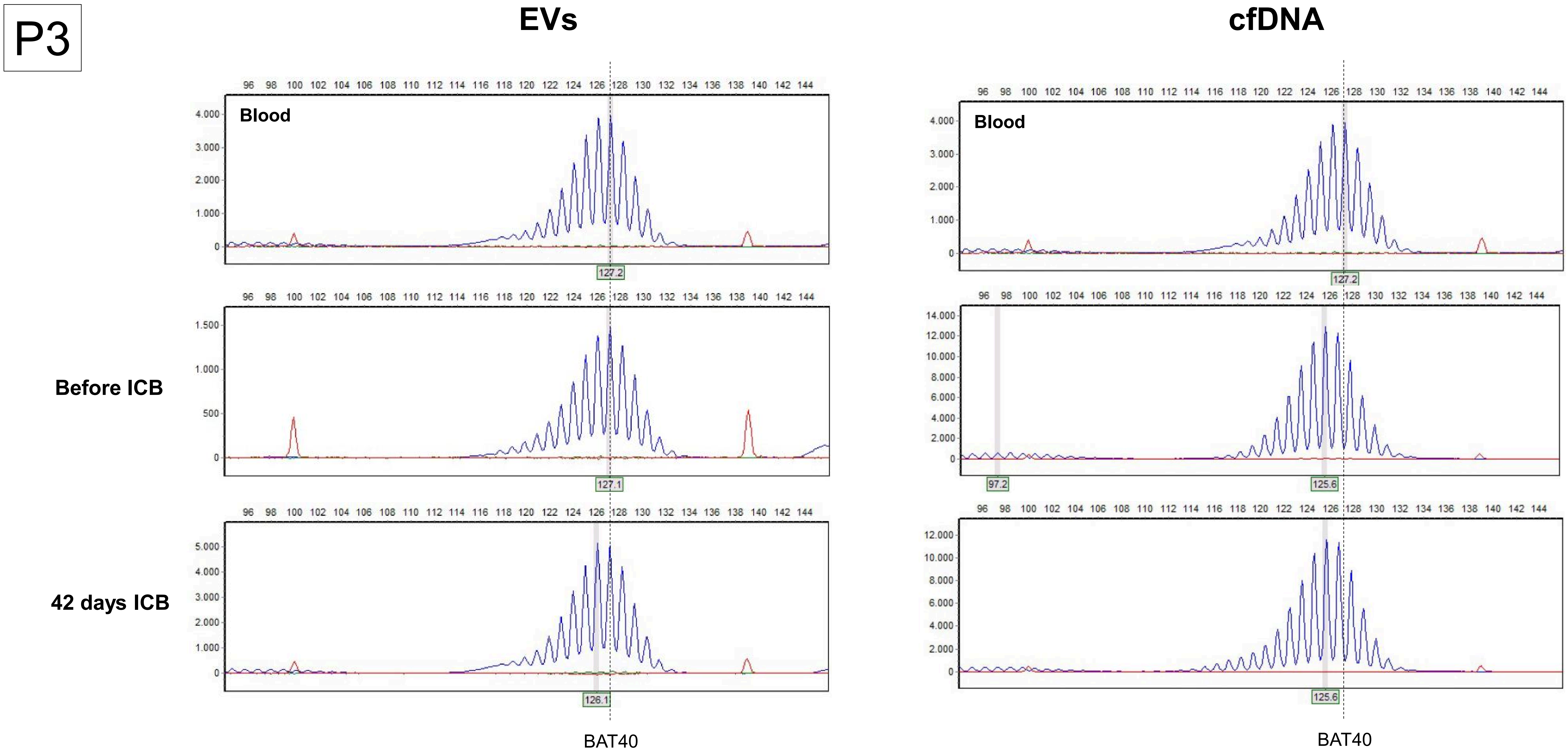
P3
EVs
cfDNA
Blood
Blood
Before ICB
42 days ICB
127.2
127.1
126.1
127.2
97.2
125.6
125.6
BAT40
BAT40

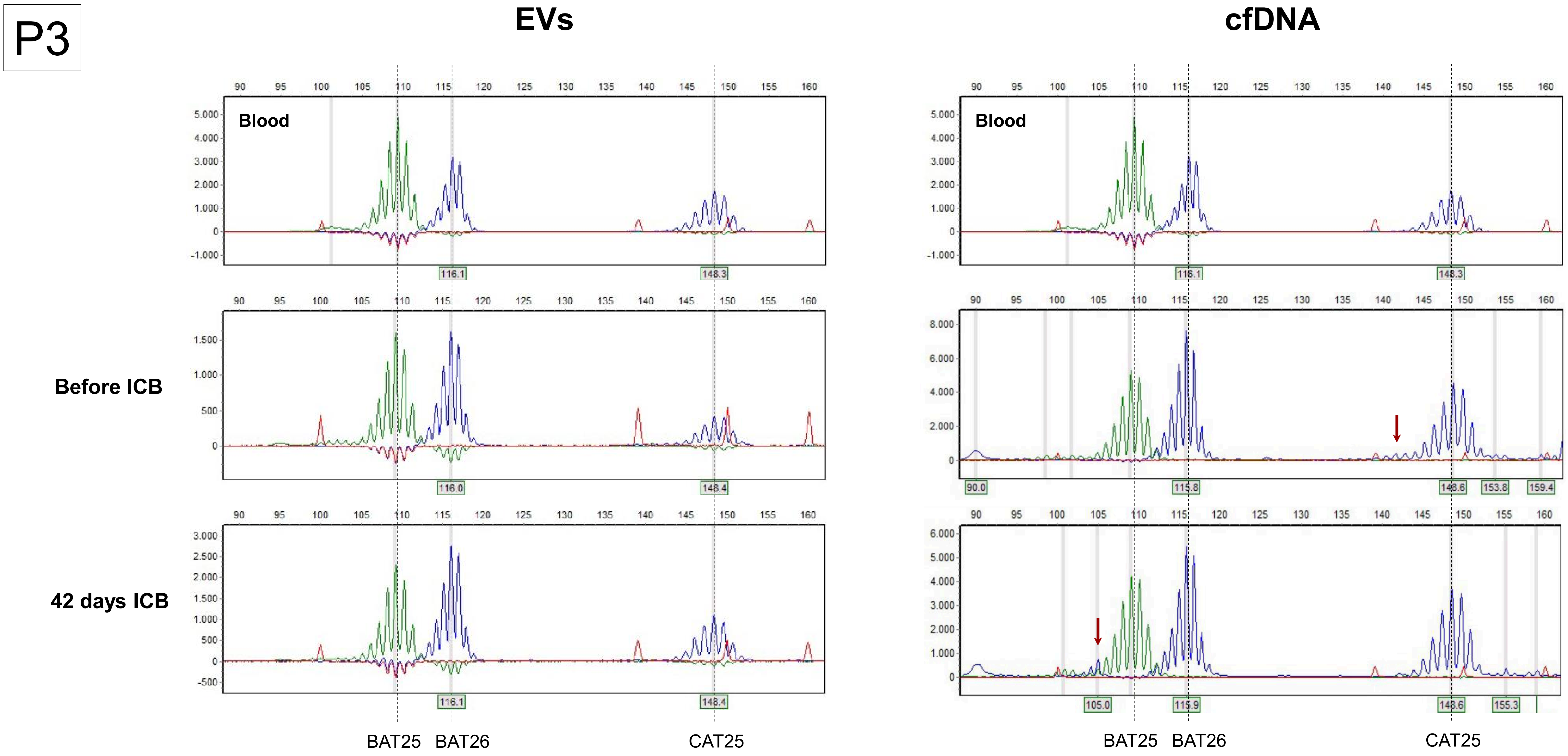

P3
EVs
cfDNA
Blood
Blood
Before ICB
42 days ICB
BAT25
BAT26
CAT25
BAT25
BAT26
CAT25

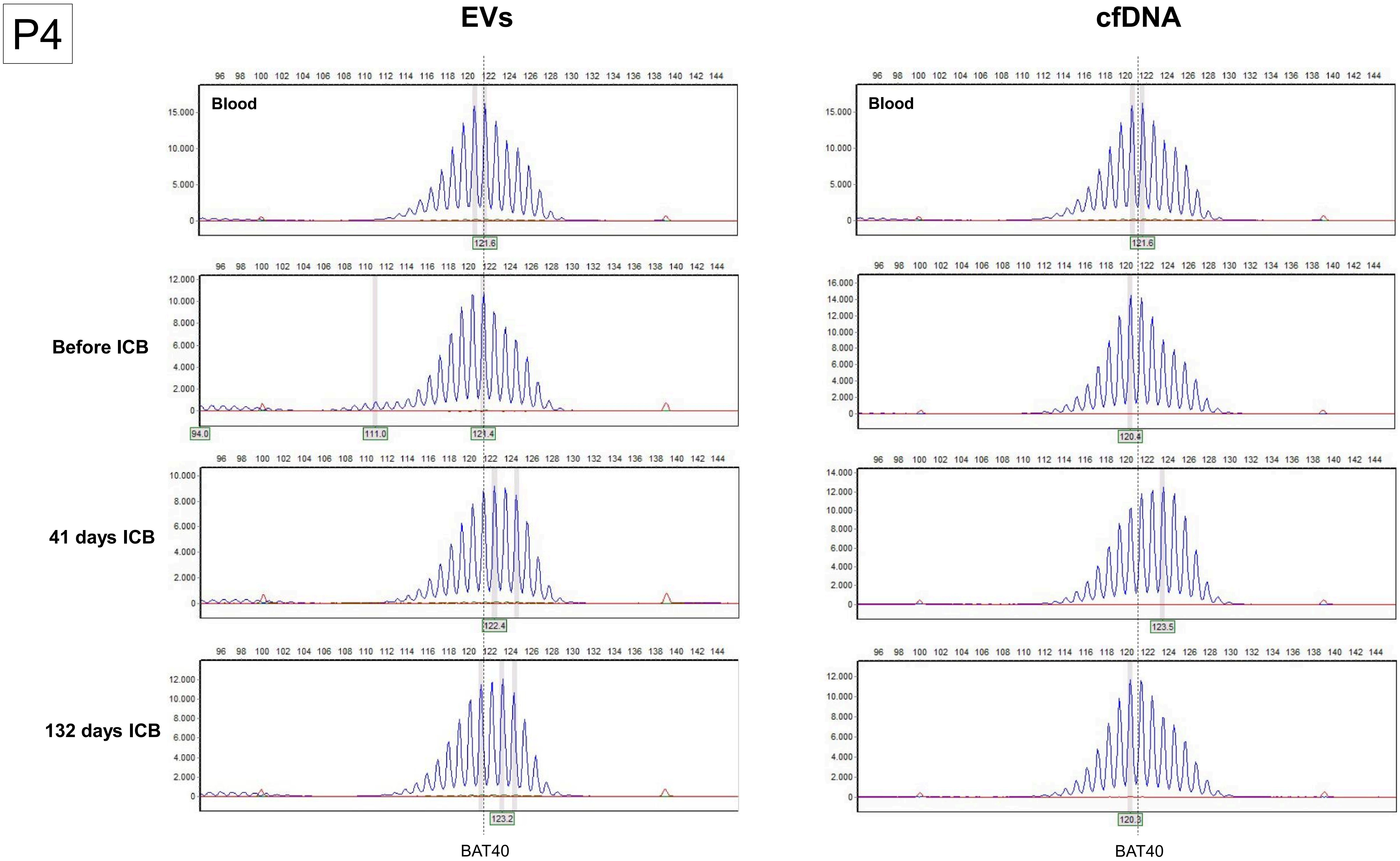

P4
EVs
cfDNA
Blood
Before ICB
41 days ICB
132 days ICB
BAT40

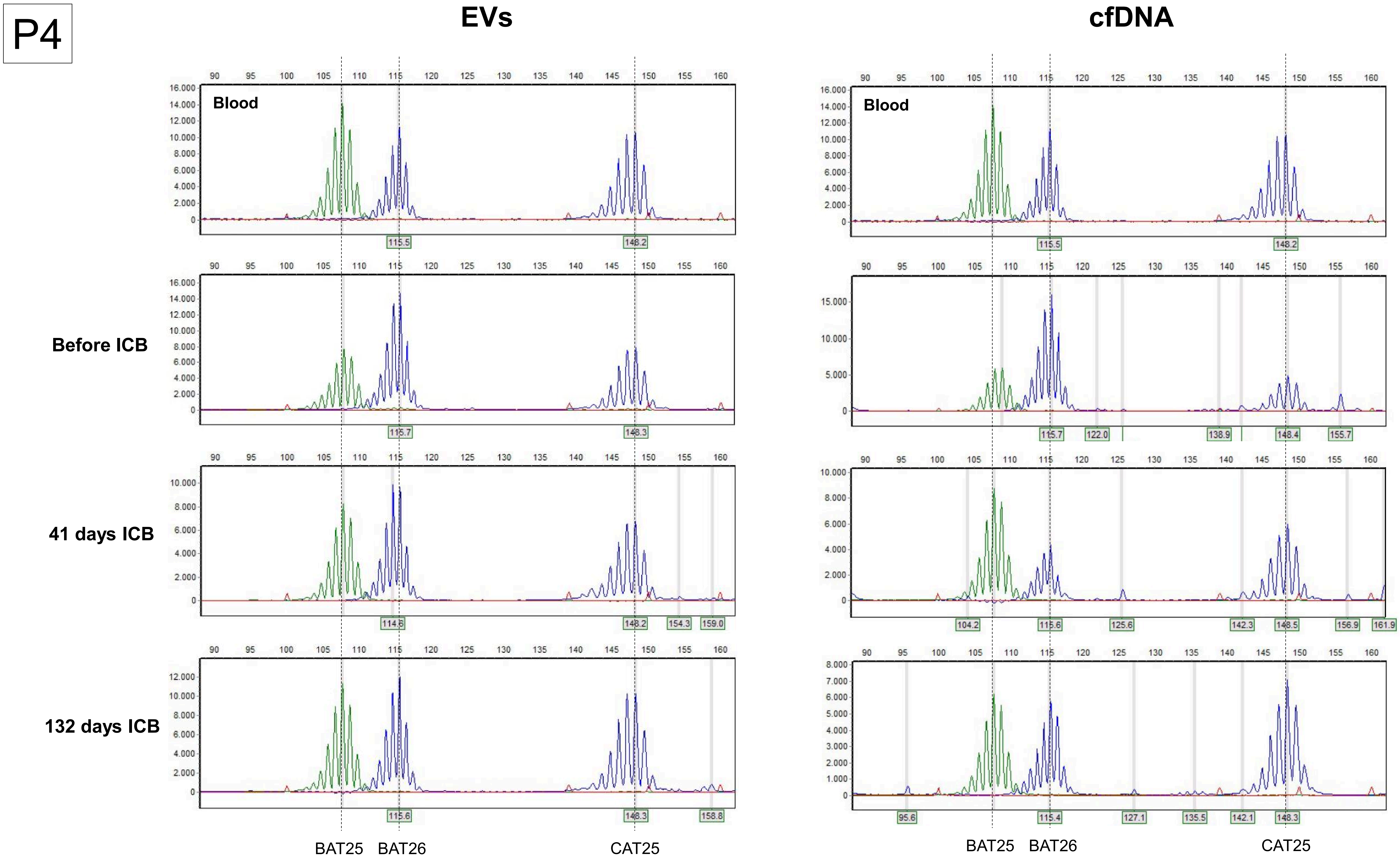
P4
EVs
cfDNA
Blood
Before ICB
41 days ICB
132 days ICB
BAT25
BAT26
CAT25

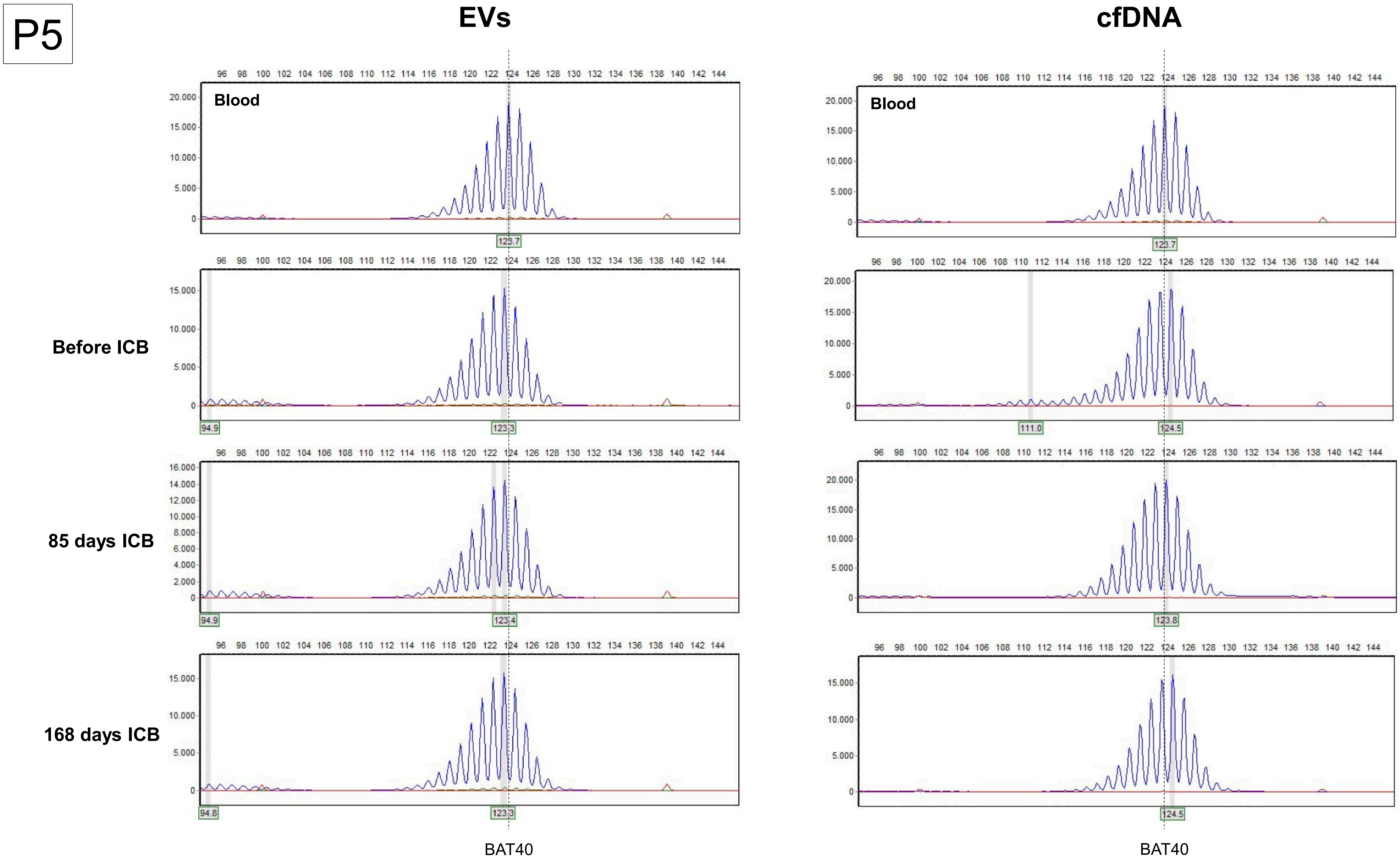
P5
EVs
cfDNA
Blood
Before ICB
85 days ICB
168 days ICB
BAT40
BAT40

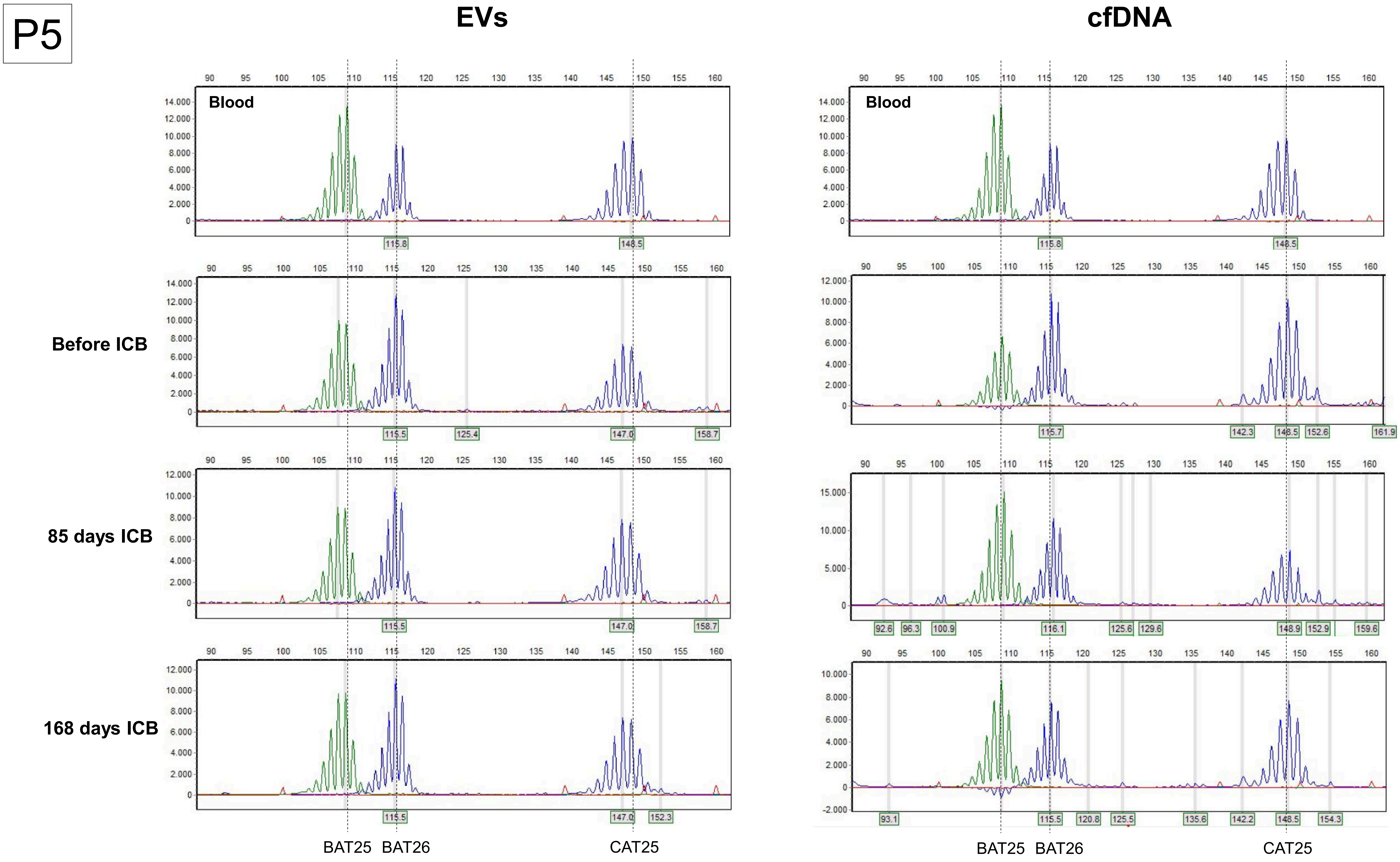
P5
EVs
cfDNA
Blood
Blood
Before ICB
85 days ICB
168 days ICB
BAT25
BAT26
CAT25
BAT25
BAT26
CAT25

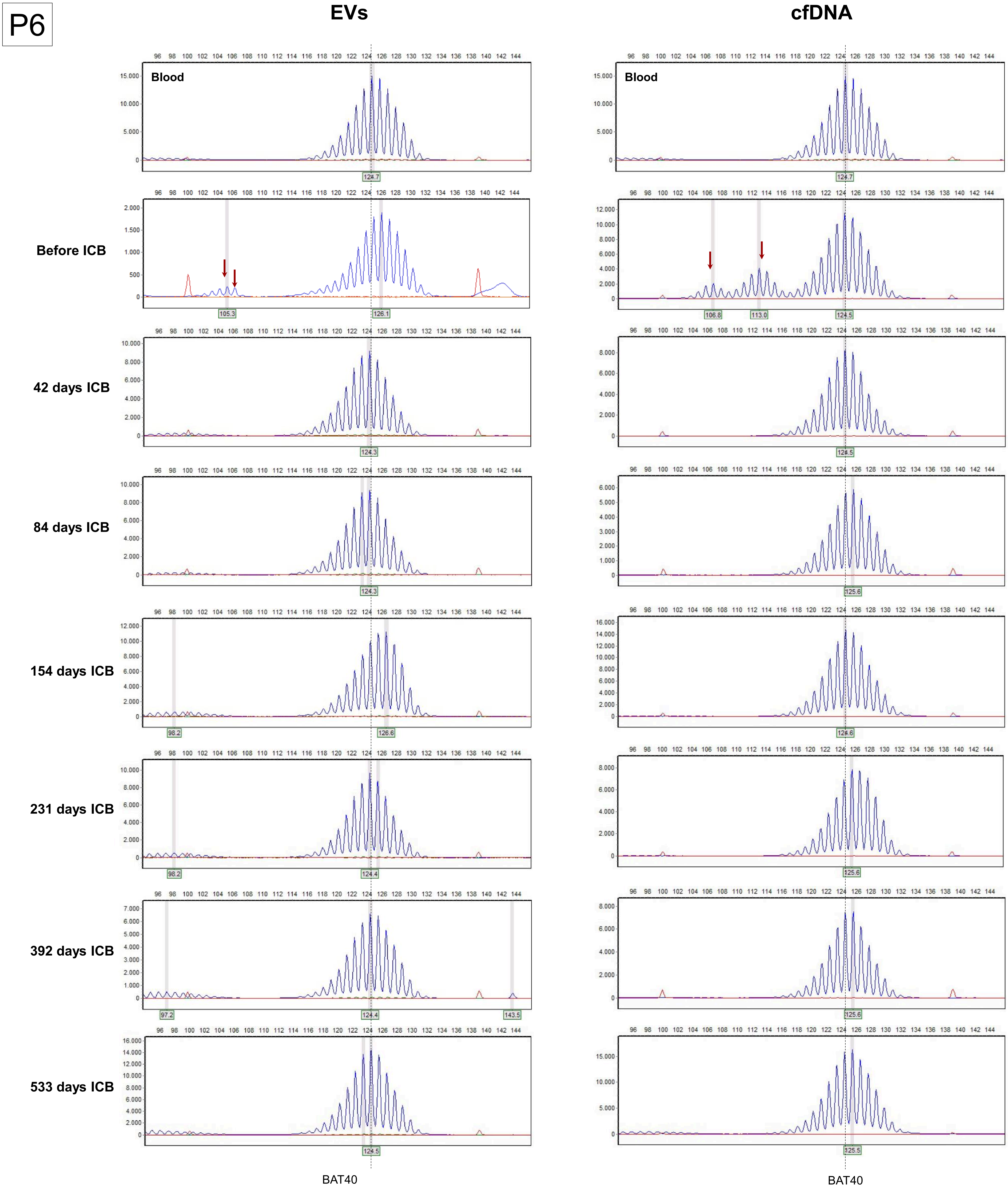
P6
EVs
cfDNA
Blood
Blood
Before ICB
42 days ICB
84 days ICB
154 days ICB
231 days ICB
392 days ICB
533 days ICB
124.7
124.7
105.3
126.1
106.8
113.0
124.5
124.3
124.5
124.3
125.6
98.2
126.6
124.6
98.2
124.4
125.6
97.2
124.4
143.5
125.6
124.5
125.5
BAT40
BAT40

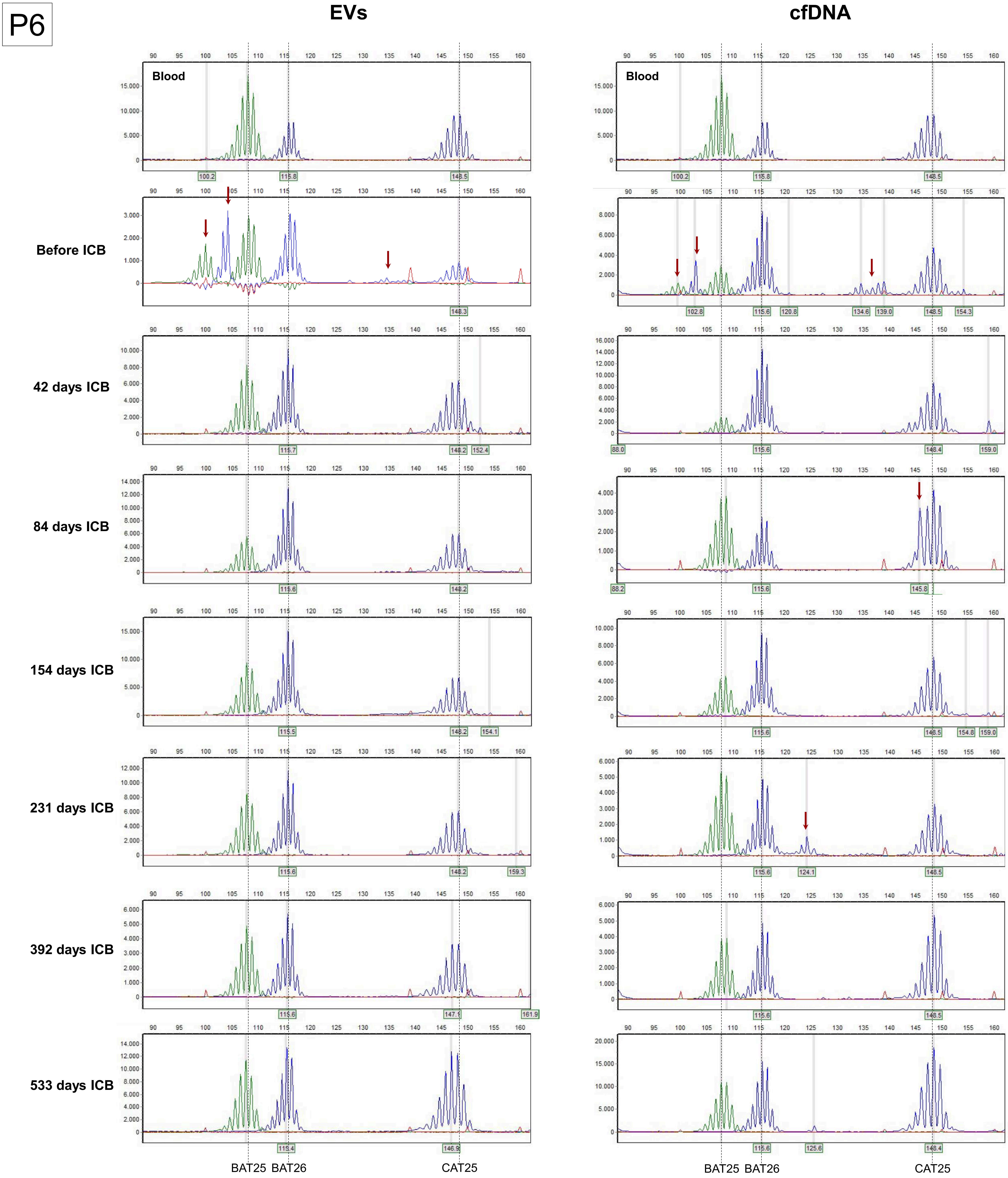
P6
EVs
cfDNA
Blood
Before ICB
42 days ICB
84 days ICB
154 days ICB
231 days ICB
392 days ICB
533 days ICB
BAT25 BAT26
CAT25

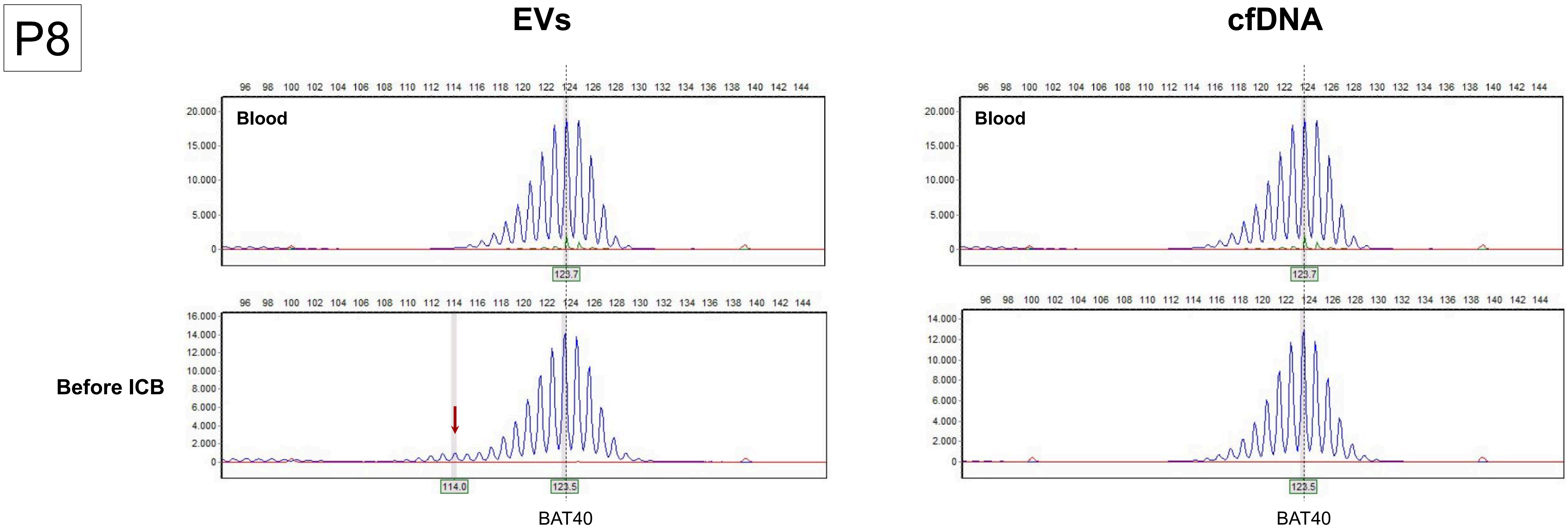
P8
EVs
cfDNA
Blood
Blood
Before ICB
123.7
123.7
114.0
123.5
123.5
BAT40
BAT40

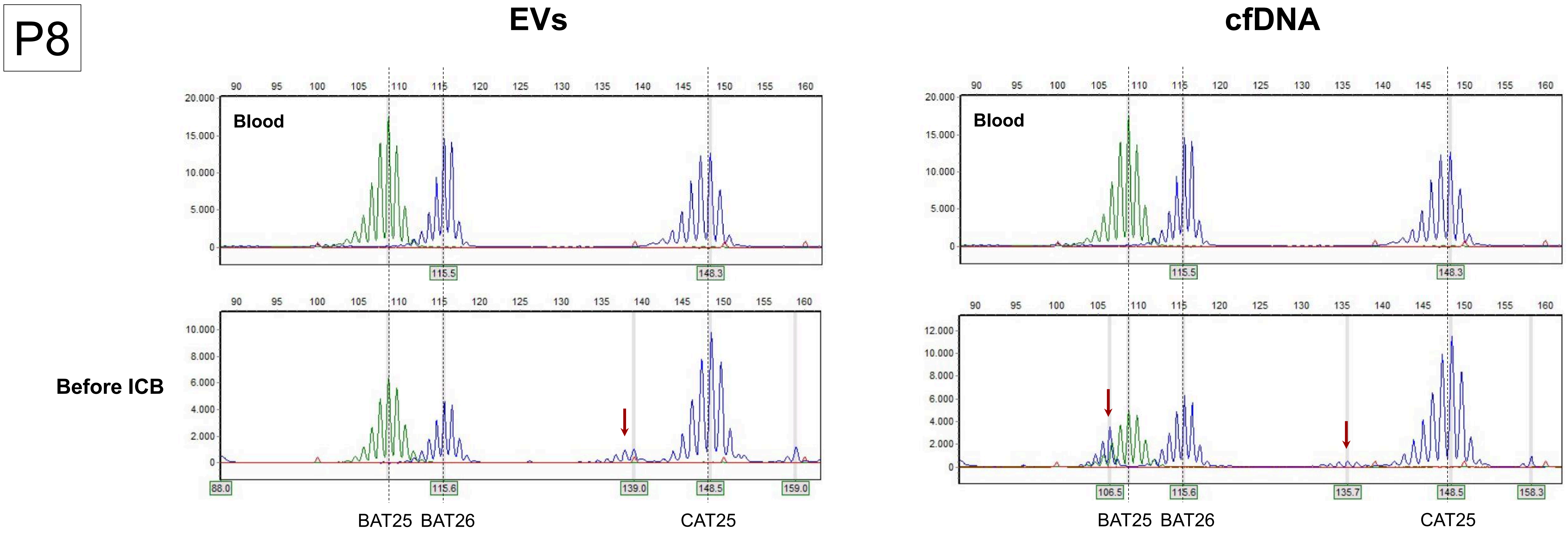
P8
EVs
cfDNA
Blood
Blood
Before ICB
BAT25 BAT26
CAT25
BAT25 BAT26
CAT25

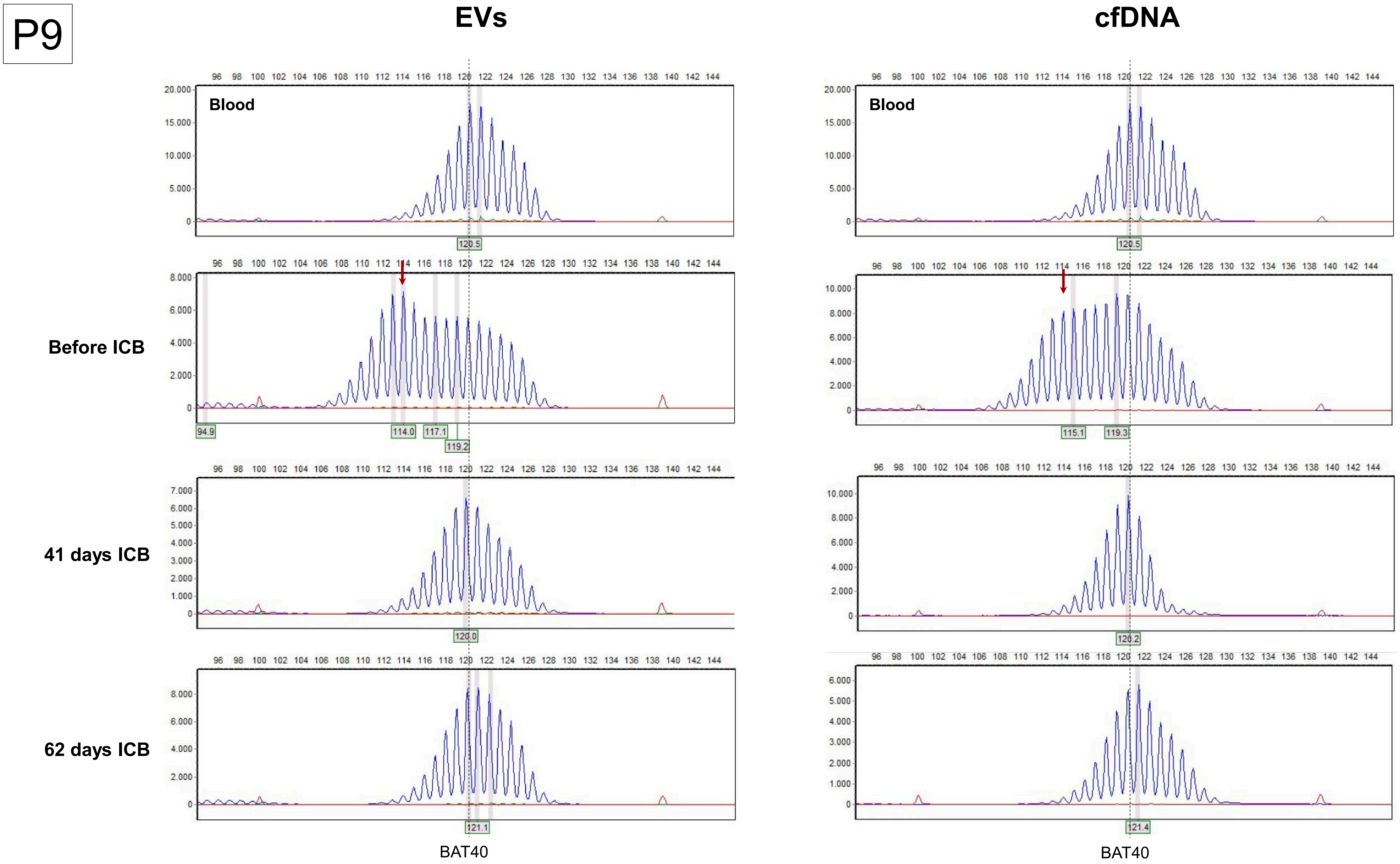

P9
EVs
cfDNA
Blood
Blood
Before ICB
41 days ICB
62 days ICB
BAT40
BAT40

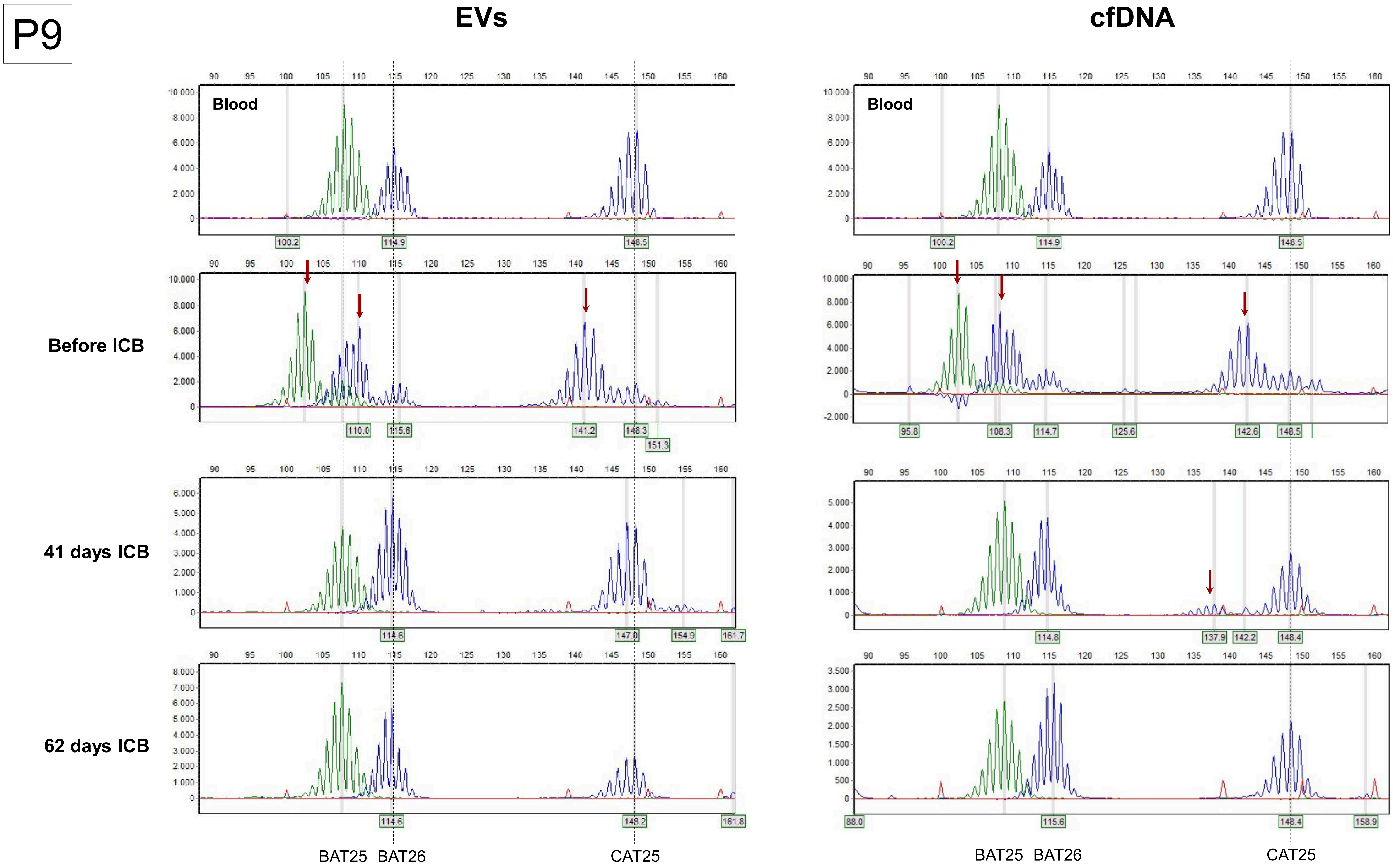

P9
EVs
cfDNA
Blood
Before ICB
41 days ICB
62 days ICB
BAT25
BAT26
CAT25

P19

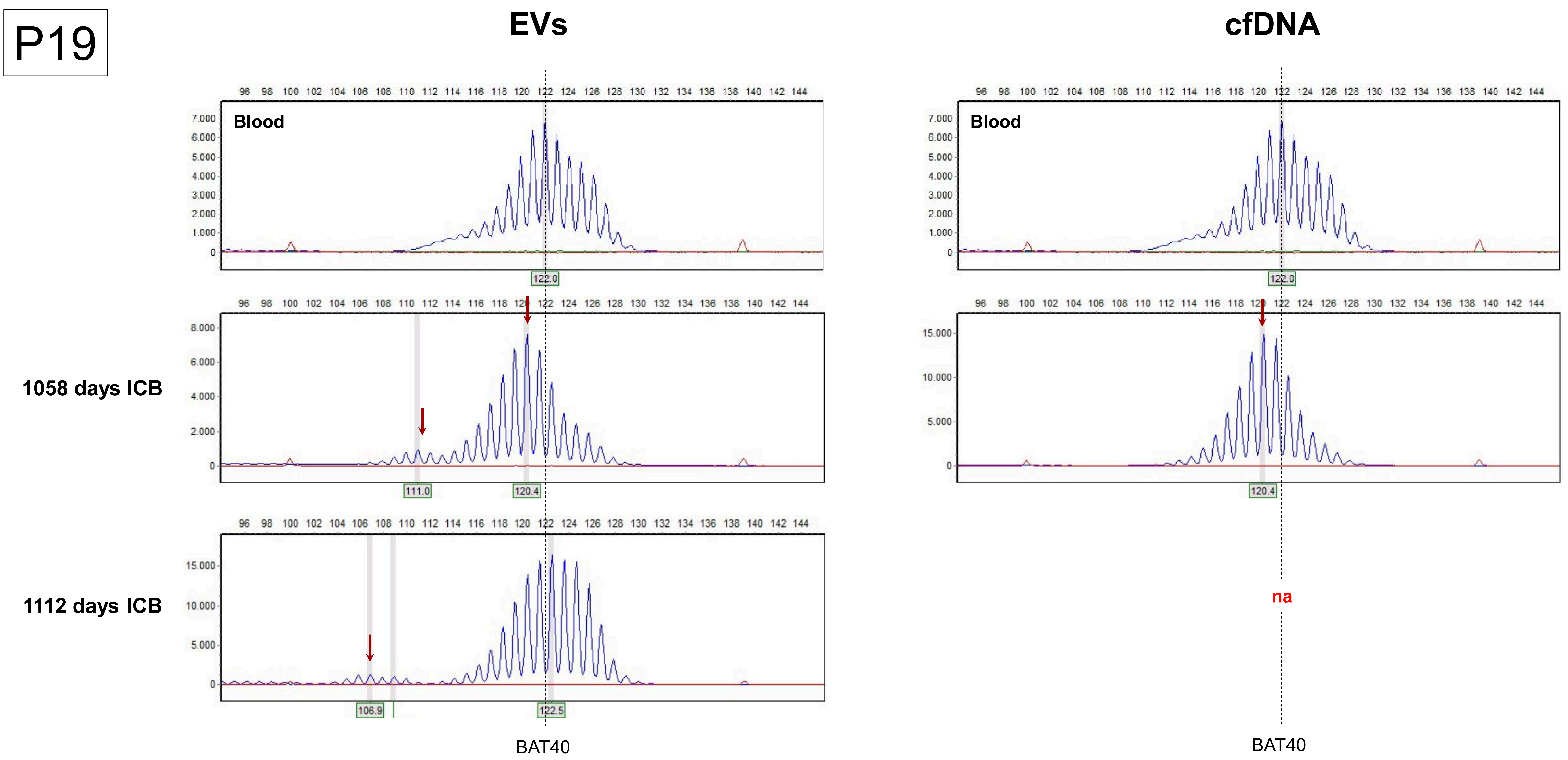
EVs
cfDNA
Blood
Blood
1058 days ICB
1112 days ICB
122.0
122.0
111.0
120.4
120.4
106.9
122.5
na
BAT40
BAT40

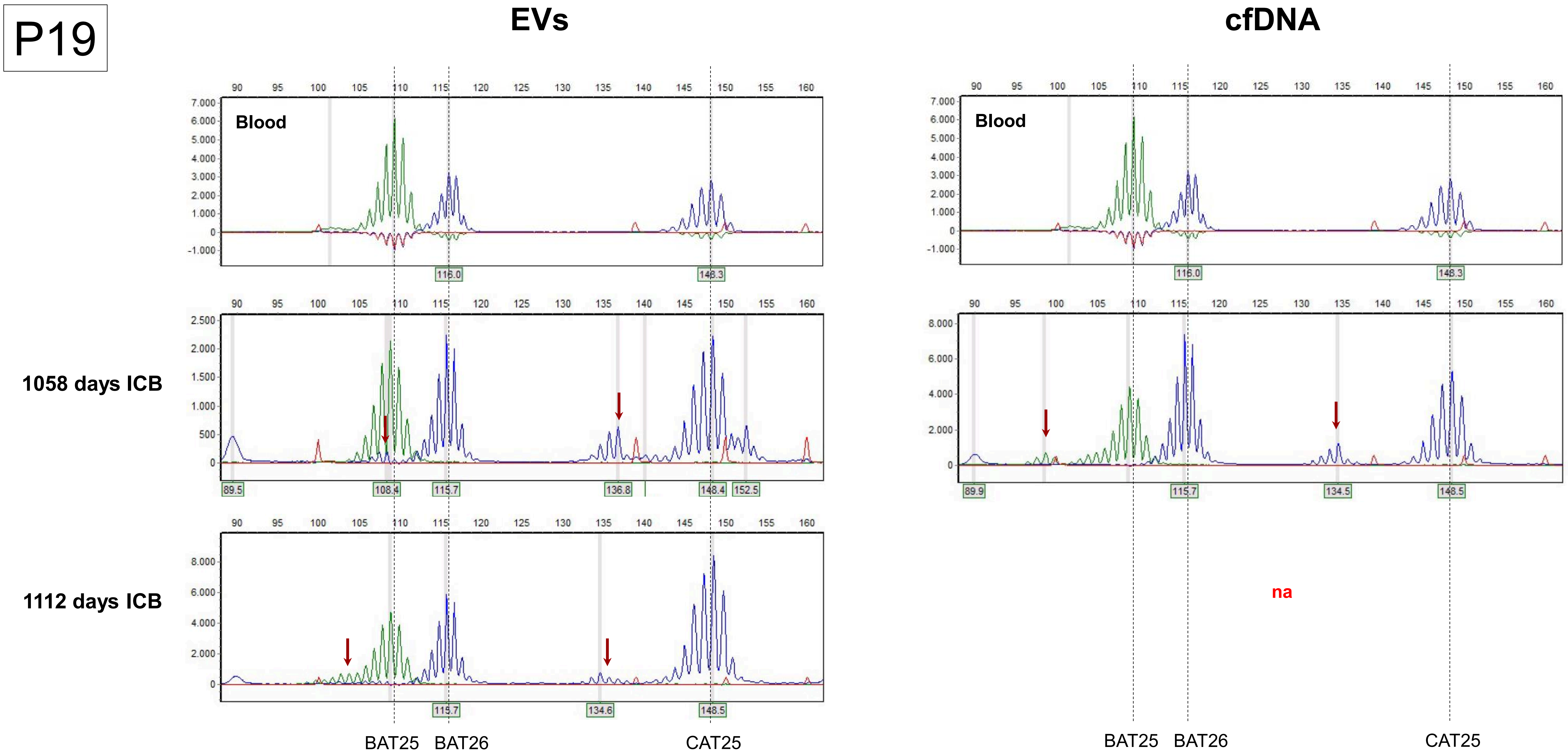
P19
EVs
cfDNA
Blood
Blood
1058 days ICB
1112 days ICB
BAT25 BAT26 CAT25
BAT25 BAT26 CAT25
na

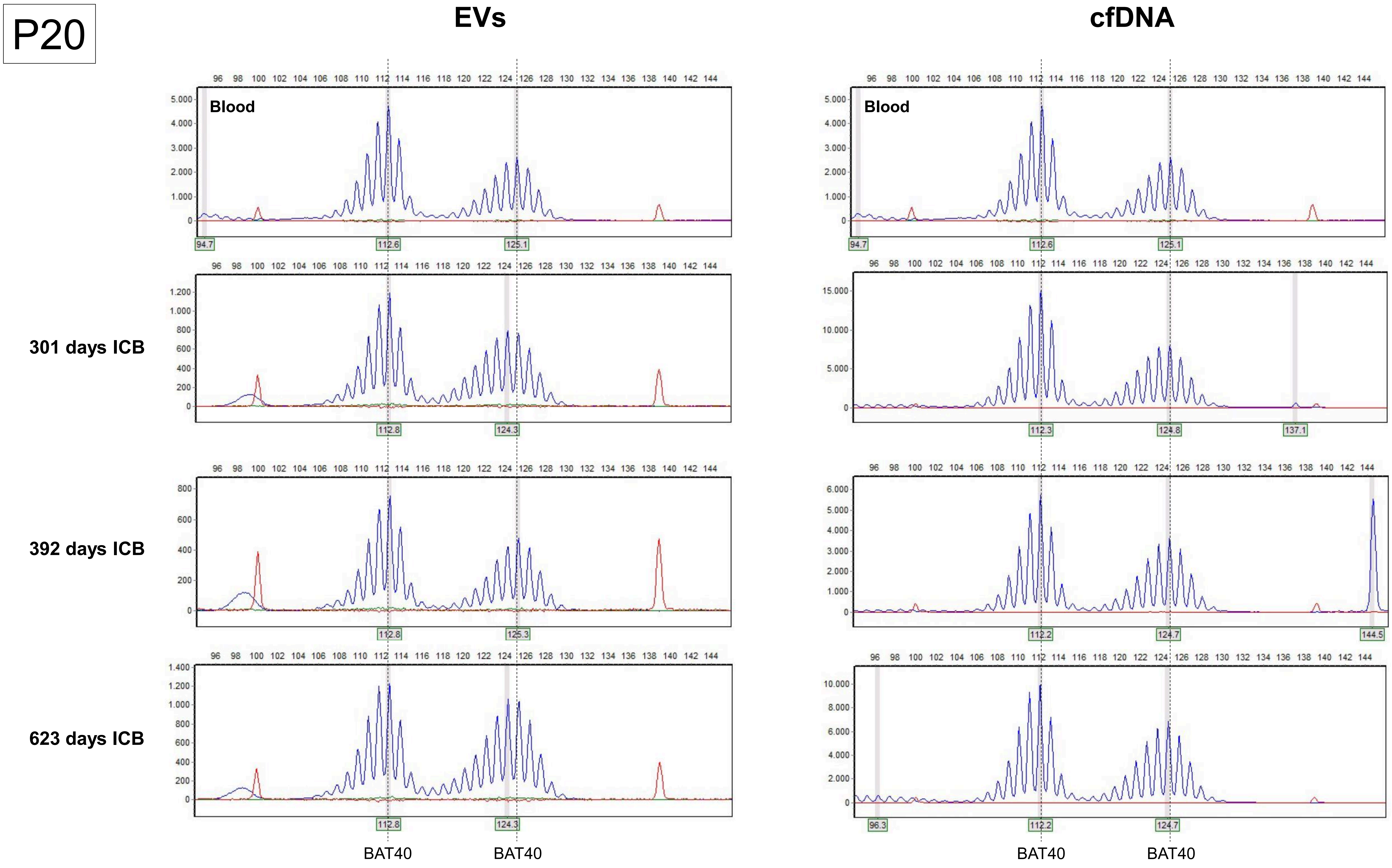
P20
EVs
cfDNA
Blood
Blood
301 days ICB
392 days ICB
623 days ICB
BAT40
BAT40
BAT40
BAT40

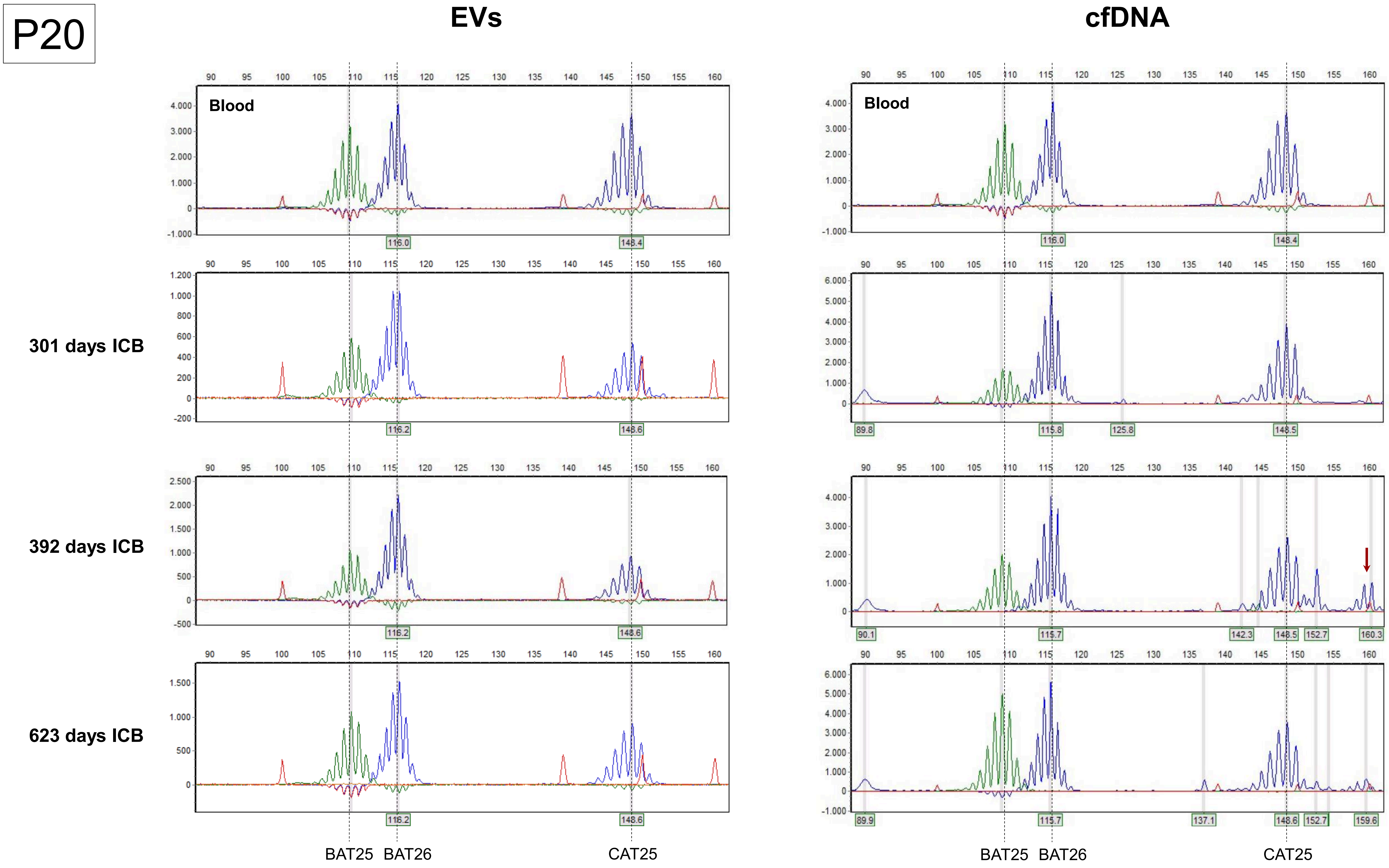
P20
EVs
cfDNA
Blood
Blood
301 days ICB
392 days ICB
623 days ICB
BAT25 BAT26
CAT25
BAT25 BAT26
CAT25

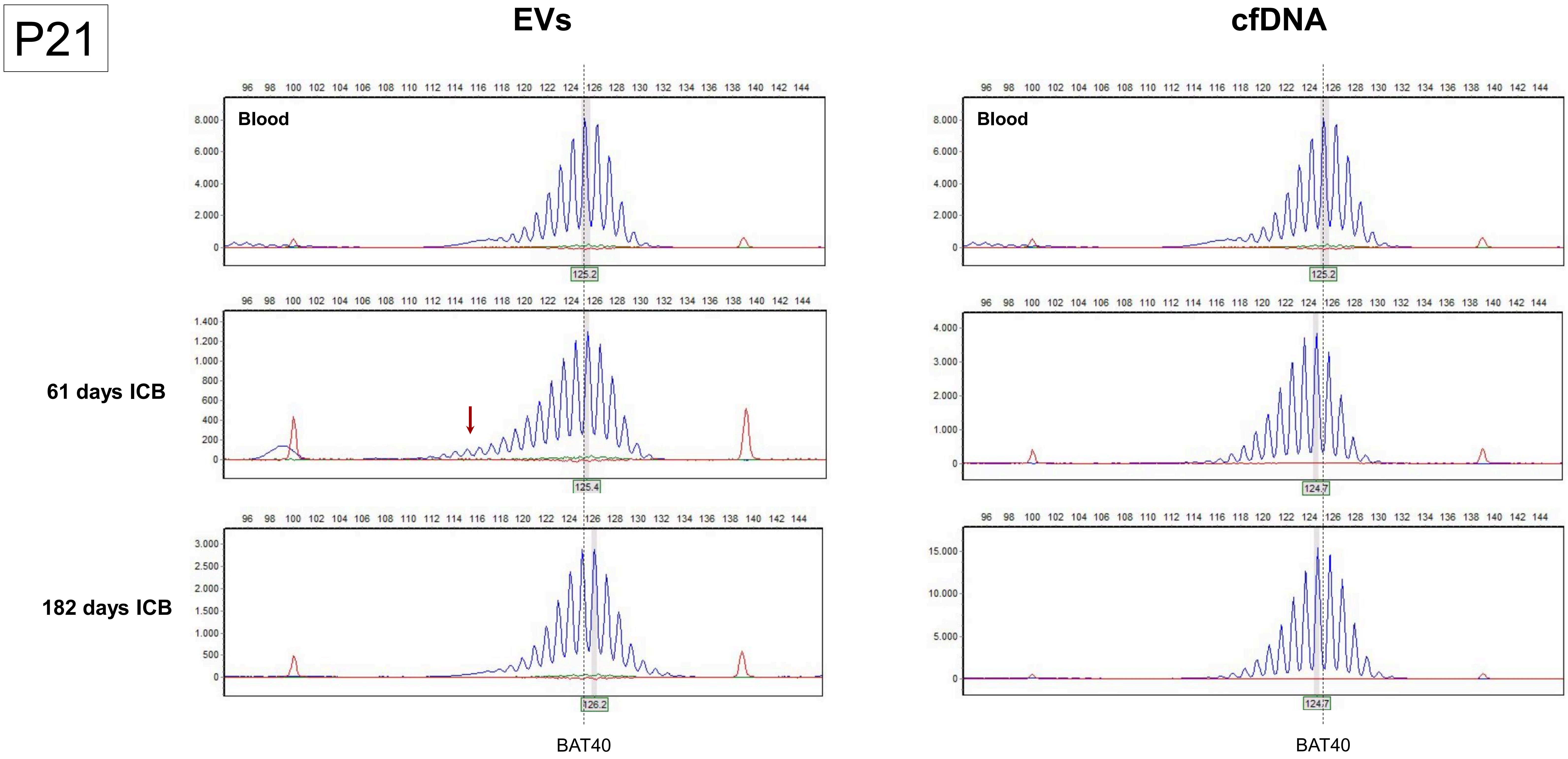
P21
EVs
cfDNA
Blood
Blood
61 days ICB
182 days ICB
125.2
125.2
125.4
124.7
126.2
124.7
BAT40
BAT40

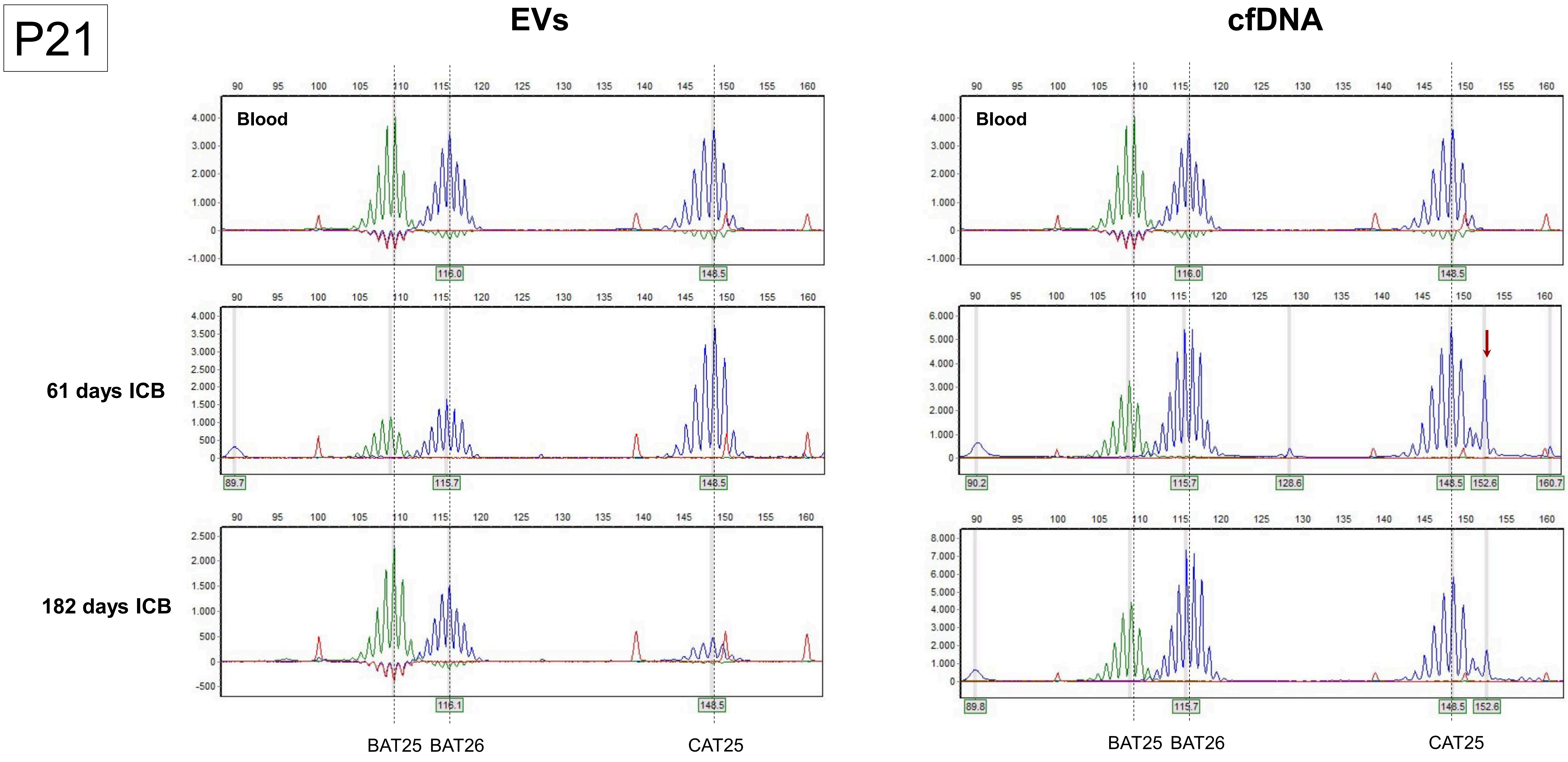
P21
EVs
cfDNA
Blood
Blood
61 days ICB
182 days ICB
BAT25 BAT26
CAT25
BAT25 BAT26
CAT25

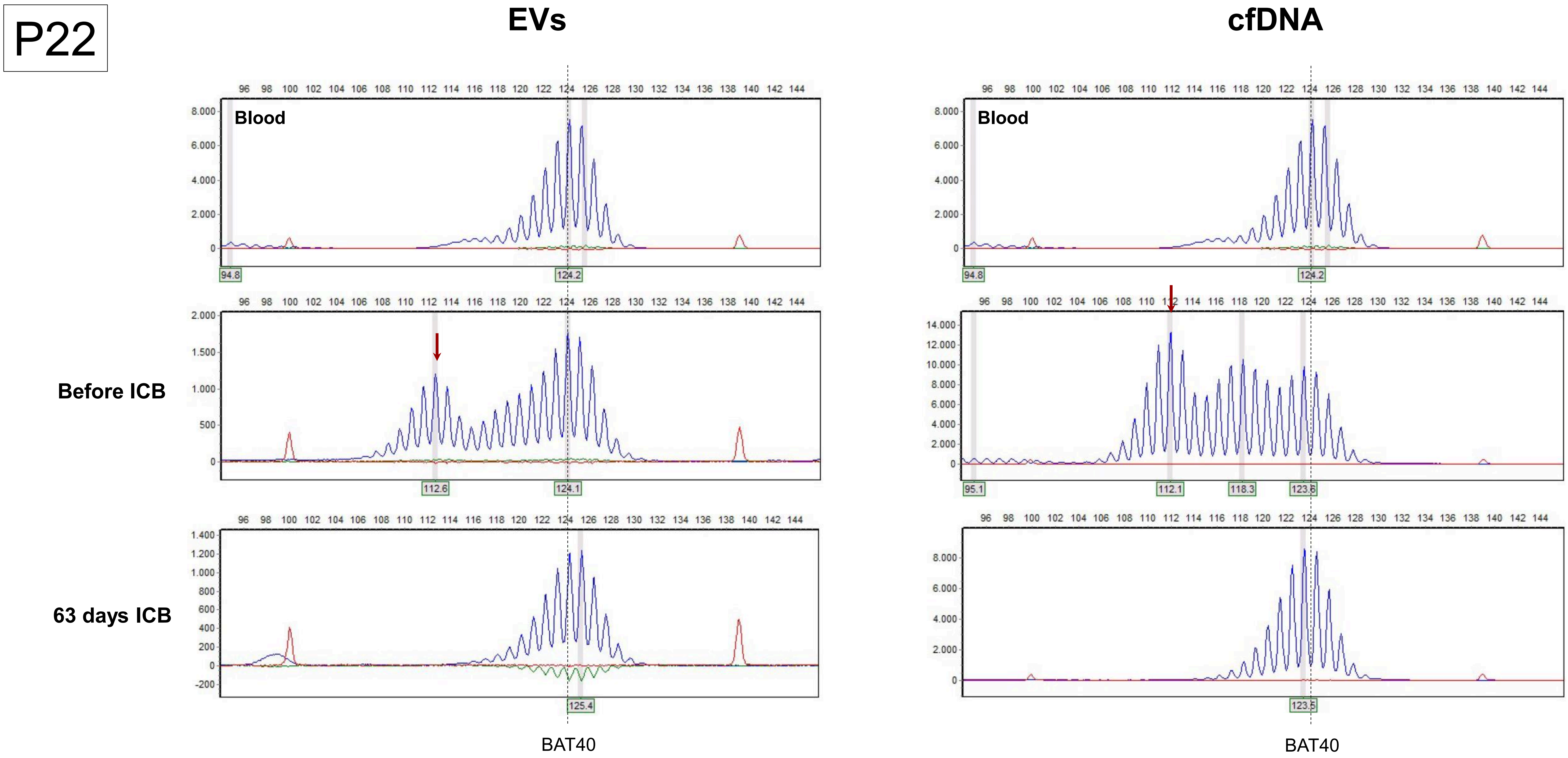
P22
EVs
cfDNA
Blood
Blood
Before ICB
63 days ICB
94.8
124.2
112.6
124.1
125.4
95.1
112.1
118.3
123.6
123.5
BAT40
BAT40

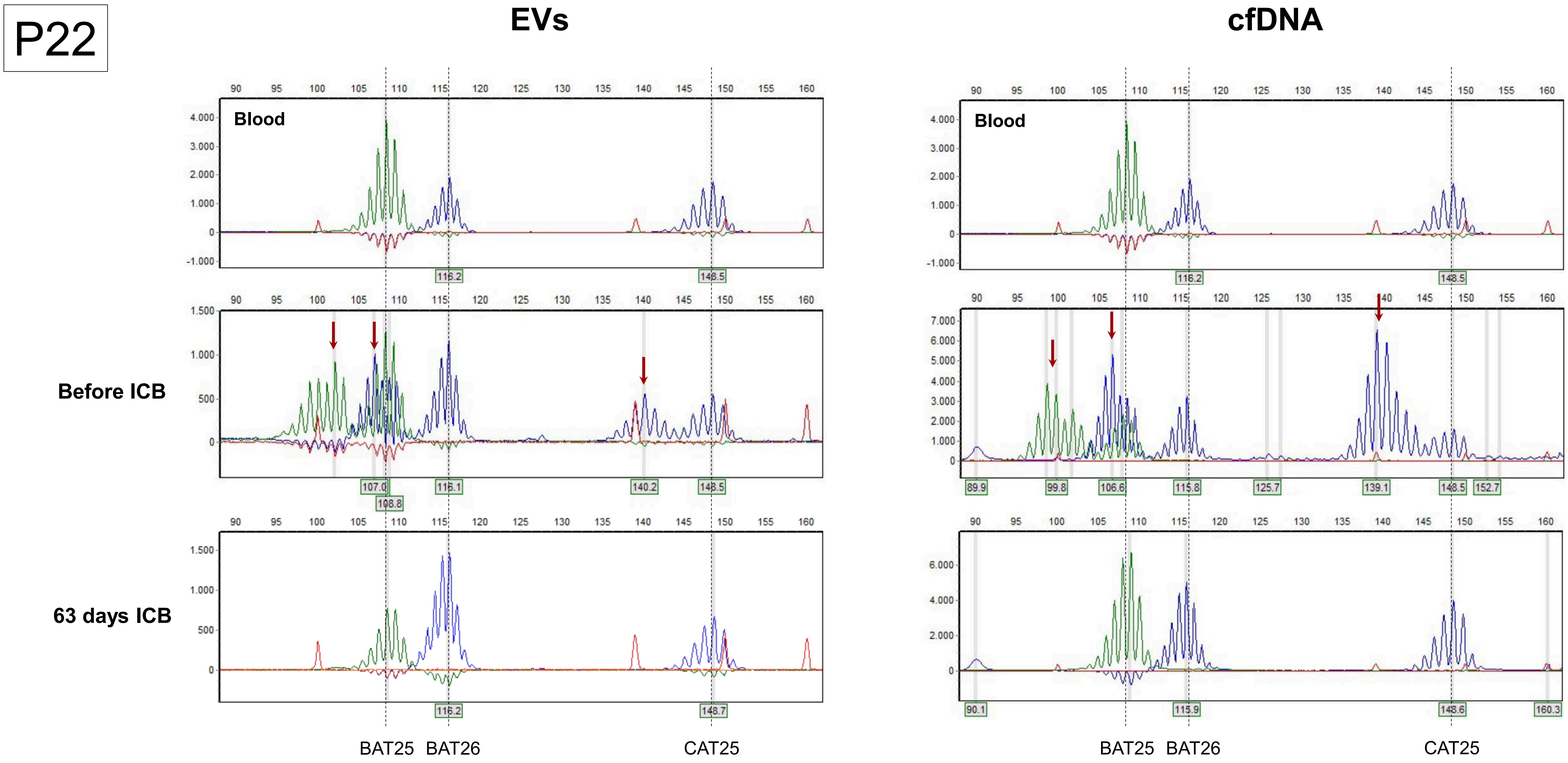
P22
EVs
cfDNA
Blood
Blood
Before ICB
63 days ICB
BAT25 BAT26
CAT25
BAT25 BAT26
CAT25

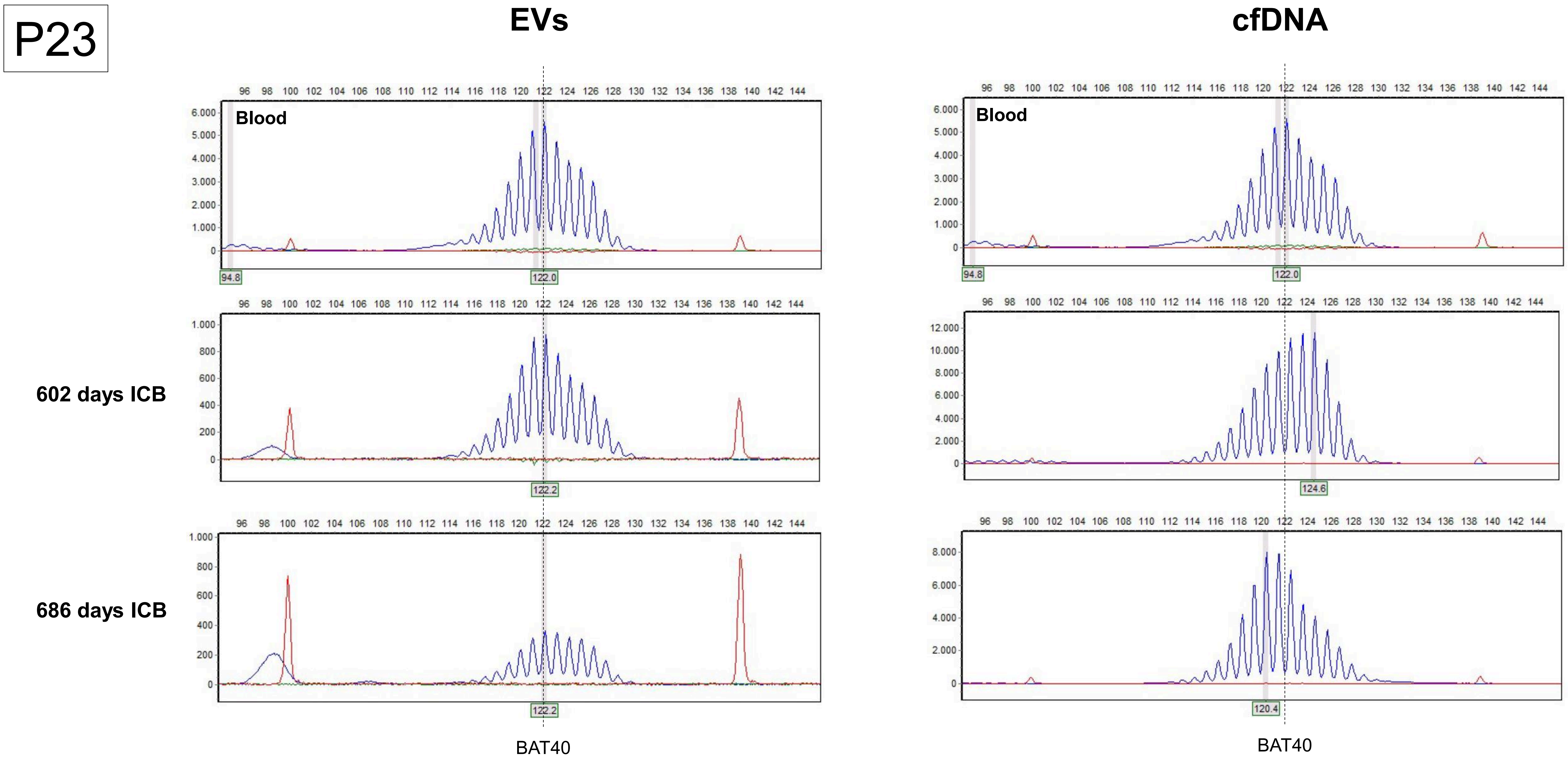

P23
EVs
cfDNA
Blood
Blood
602 days ICB
686 days ICB
BAT40
BAT40

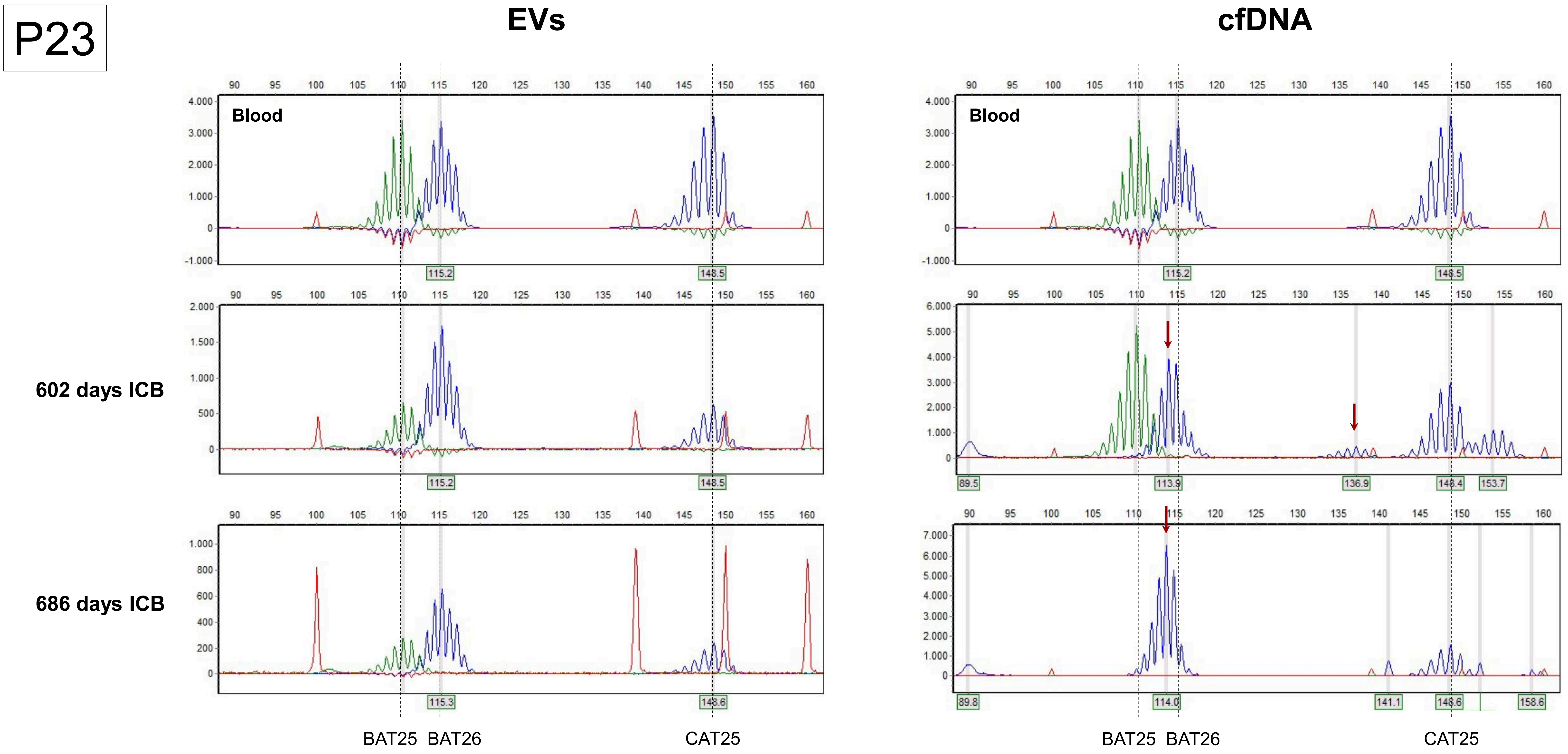
P23
EVs
cfDNA
Blood
602 days ICB
686 days ICB
BAT25 BAT26
CAT25

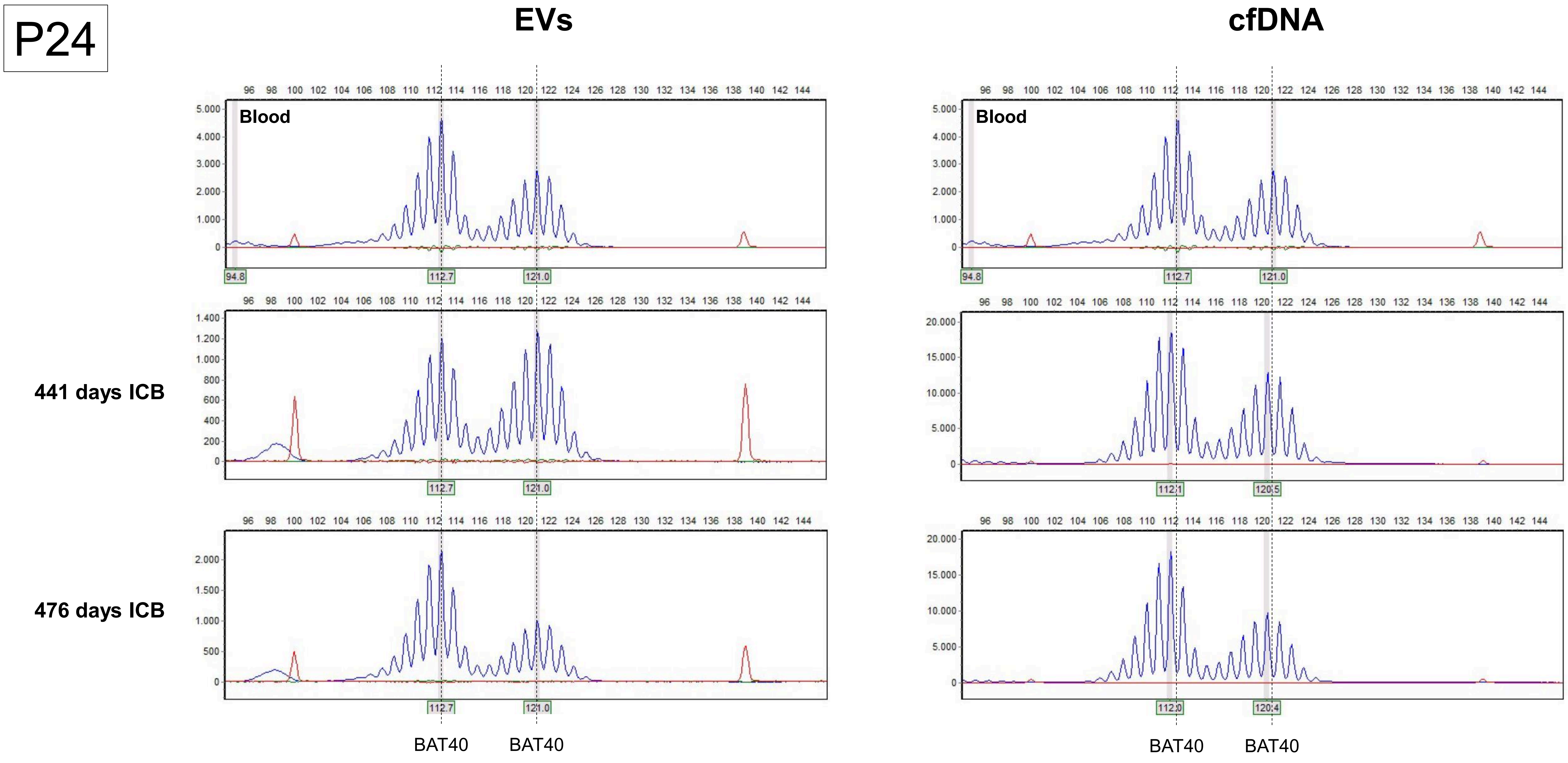

P24
EVs
cfDNA
Blood
Blood
441 days ICB
476 days ICB
BAT40
BAT40
BAT40
BAT40

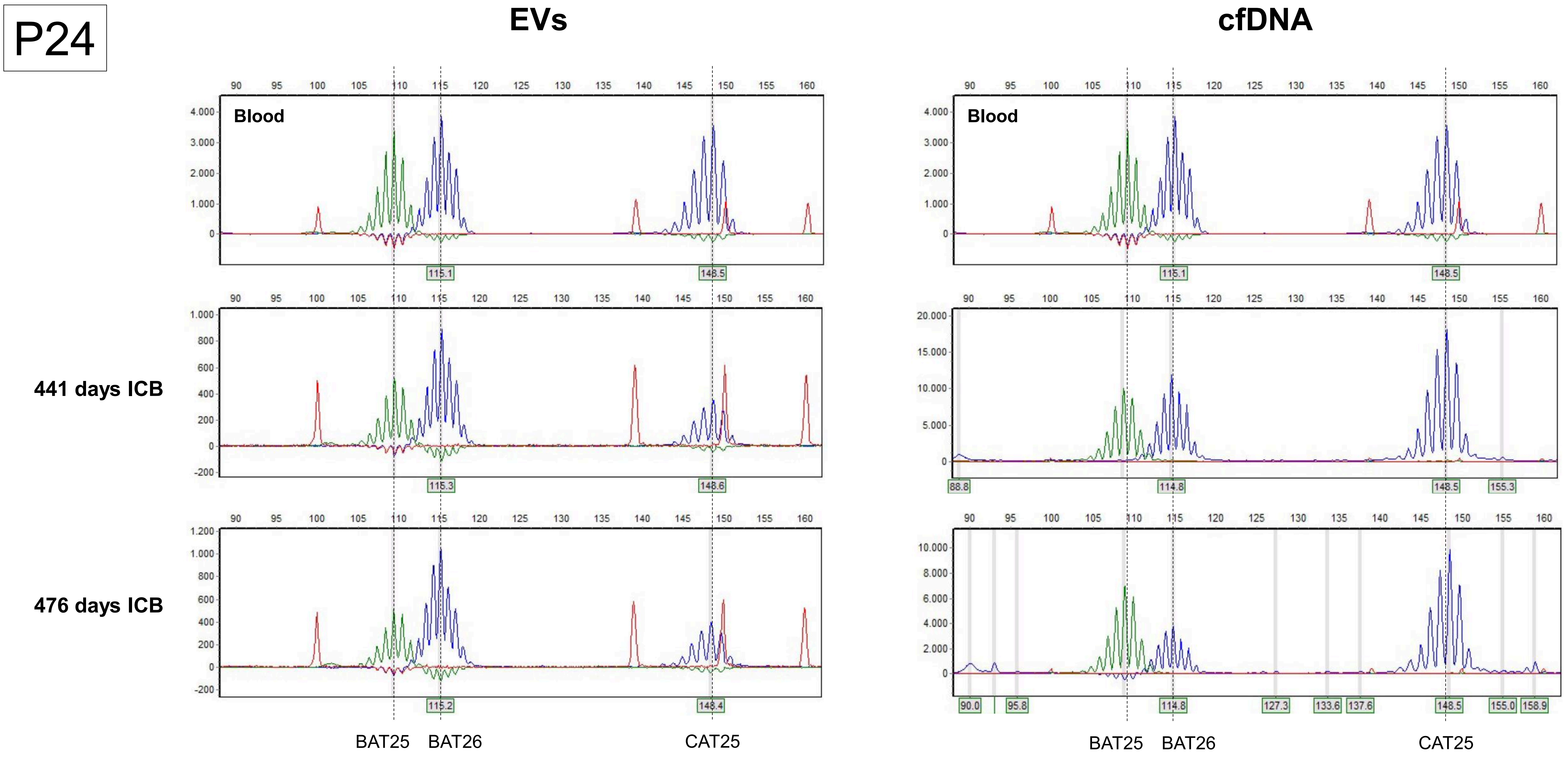
P24
EVs
cfDNA
Blood
Blood
441 days ICB
476 days ICB
115.1
148.5
115.3
148.6
115.2
148.4
115.1
148.5
88.8
114.8
148.5
155.3
90.0
95.8
114.8
127.3
133.6
137.6
148.5
155.0
158.9
BAT25
BAT26
CAT25
BAT25
BAT26
CAT25

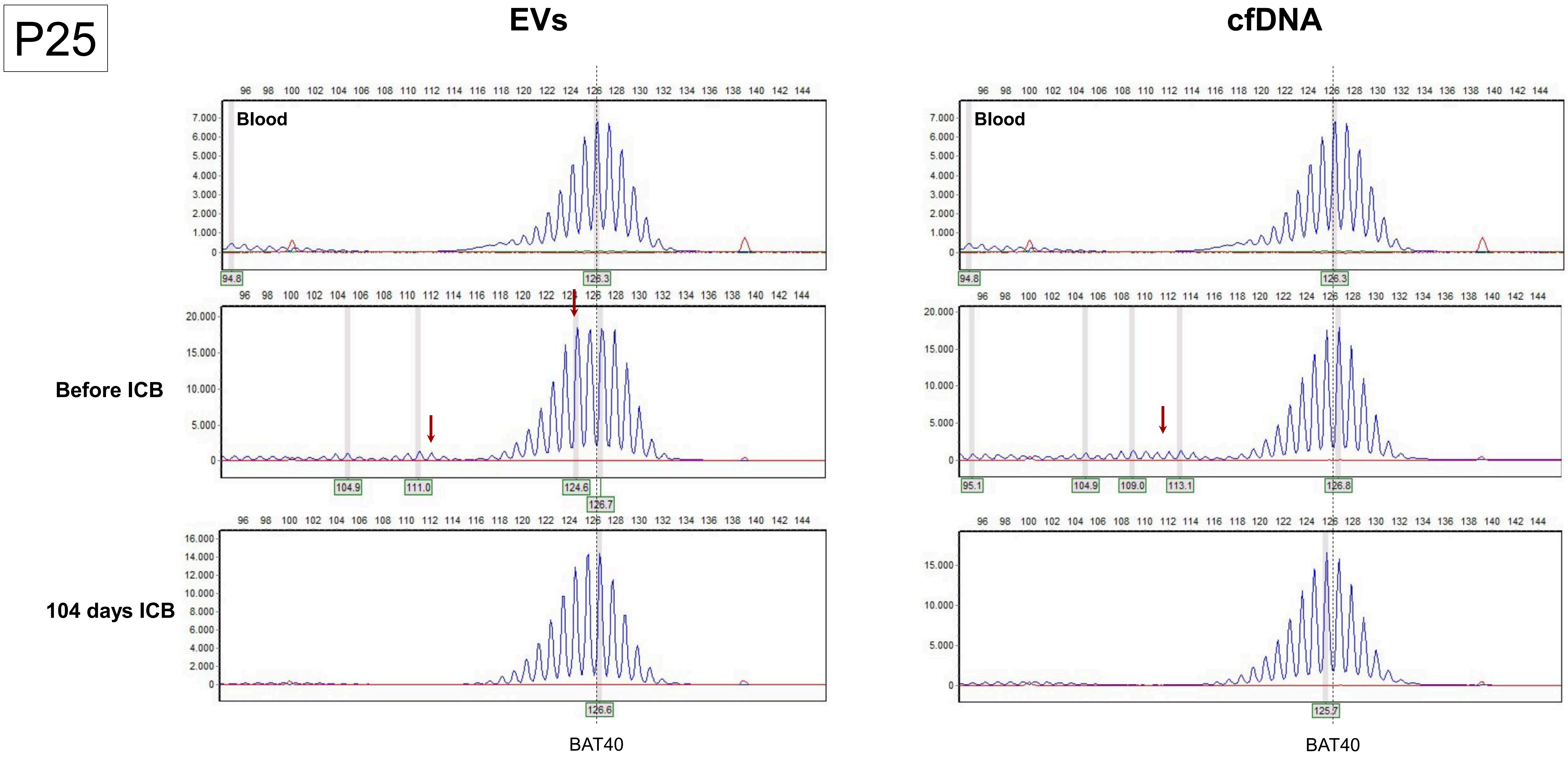
P25
EVs
cfDNA
Blood
Blood
Before ICB
104 days ICB
94.8
126.3
94.8
126.3
104.9
111.0
124.6
126.7
95.1
104.9
109.0
113.1
126.8
126.6
125.7
BAT40
BAT40

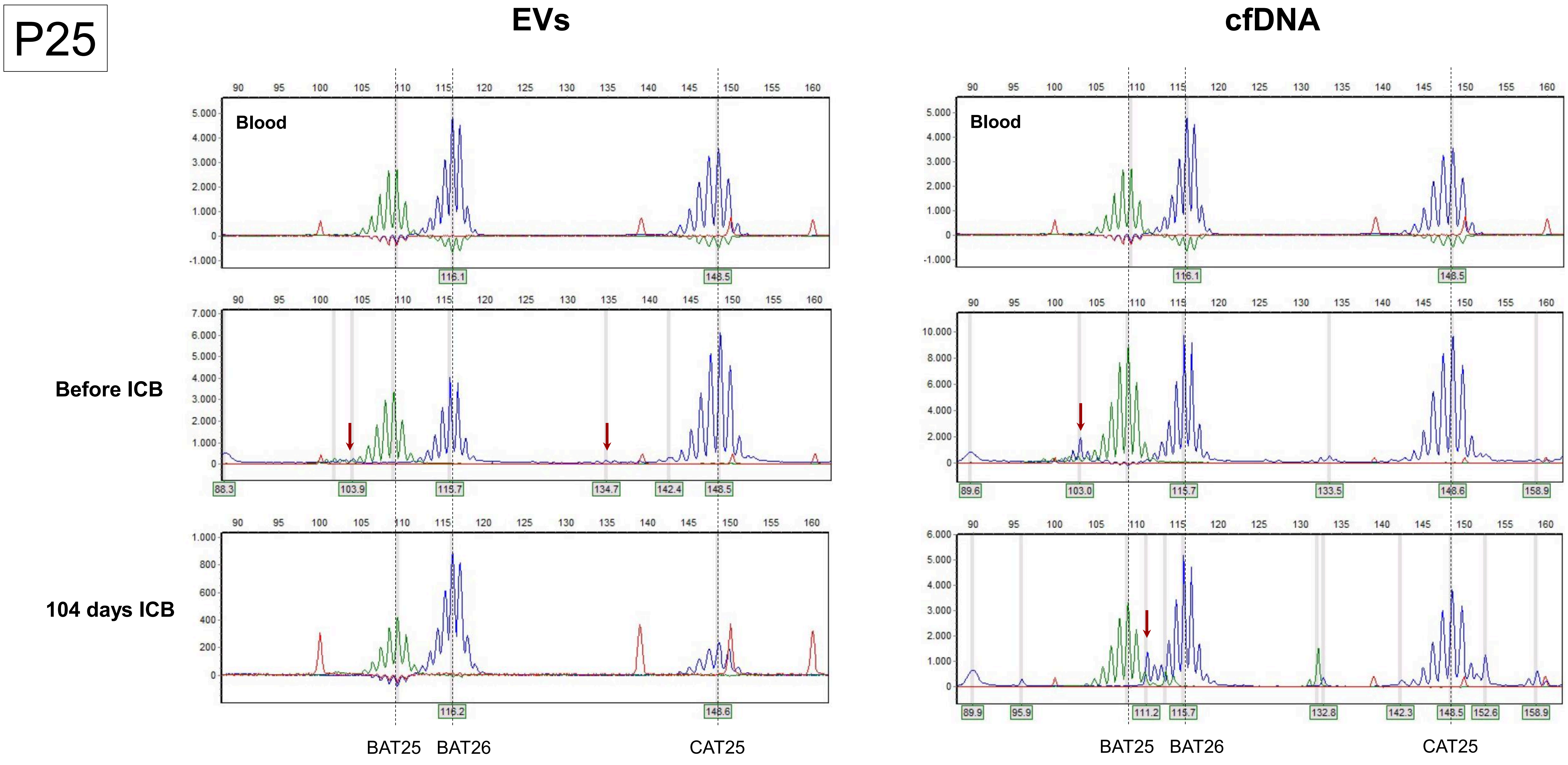
P25
EVs
cfDNA
Blood
Blood
Before ICB
104 days ICB
BAT25
BAT26
CAT25
BAT25
BAT26
CAT25

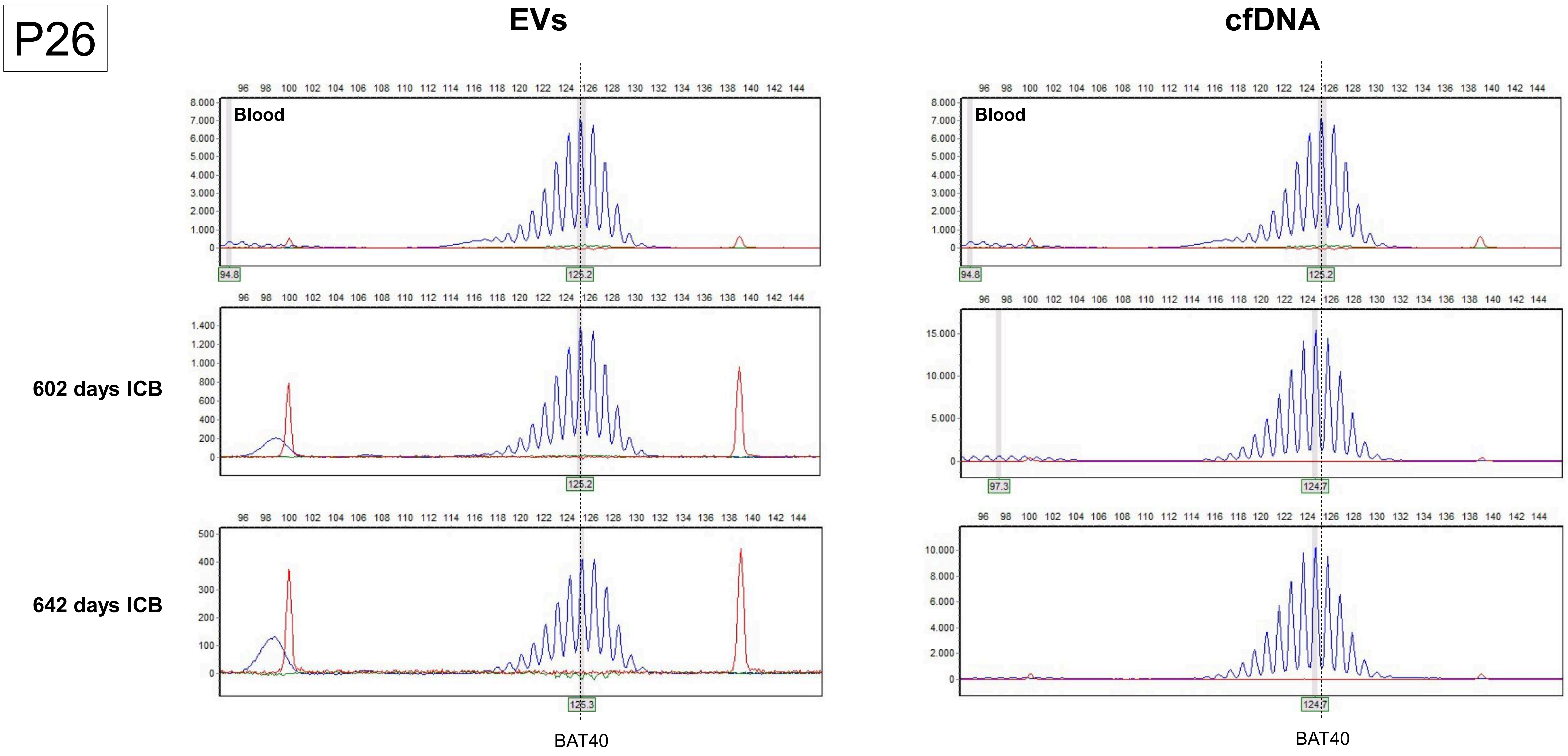
P26
EVs
cfDNA
Blood
Blood
602 days ICB
642 days ICB
BAT40
BAT40

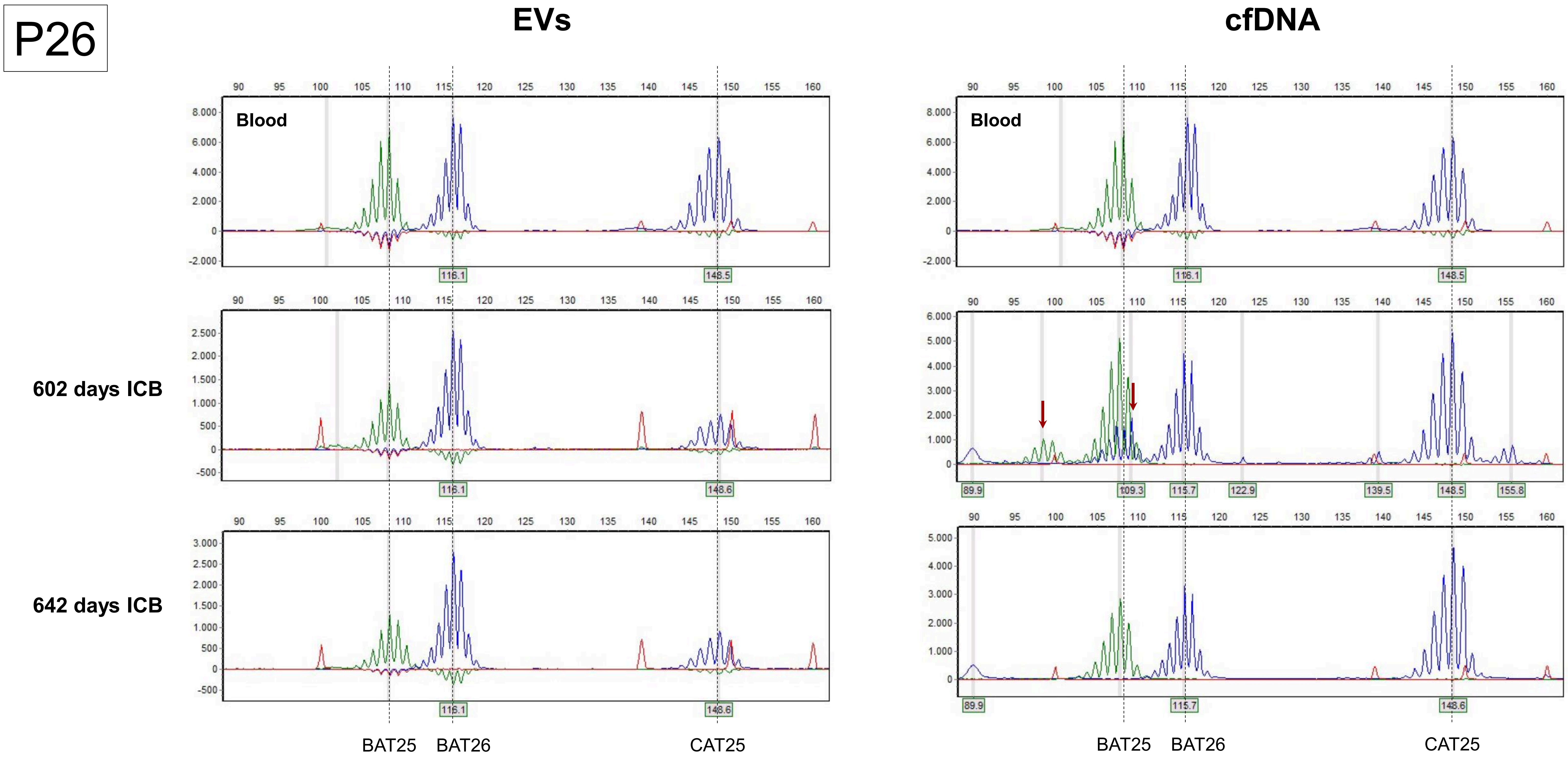
P26
EVs
cfDNA
Blood
Blood
602 days ICB
642 days ICB
BAT25
BAT26
CAT25
BAT25
BAT26
CAT25

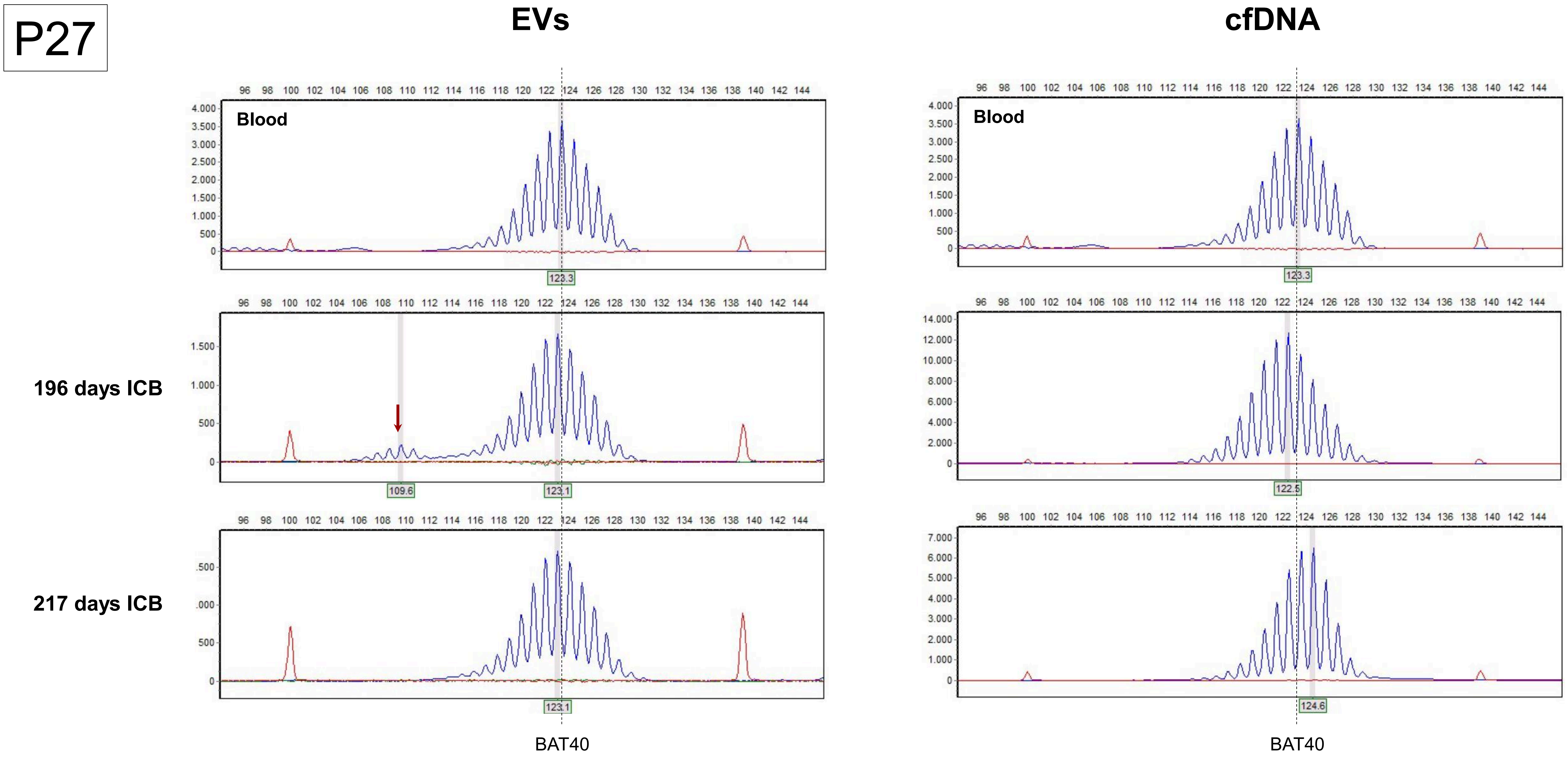
P27
EVs
cfDNA
Blood
Blood
196 days ICB
217 days ICB
109.6
123.3
123.1
122.5
124.6
BAT40
BAT40

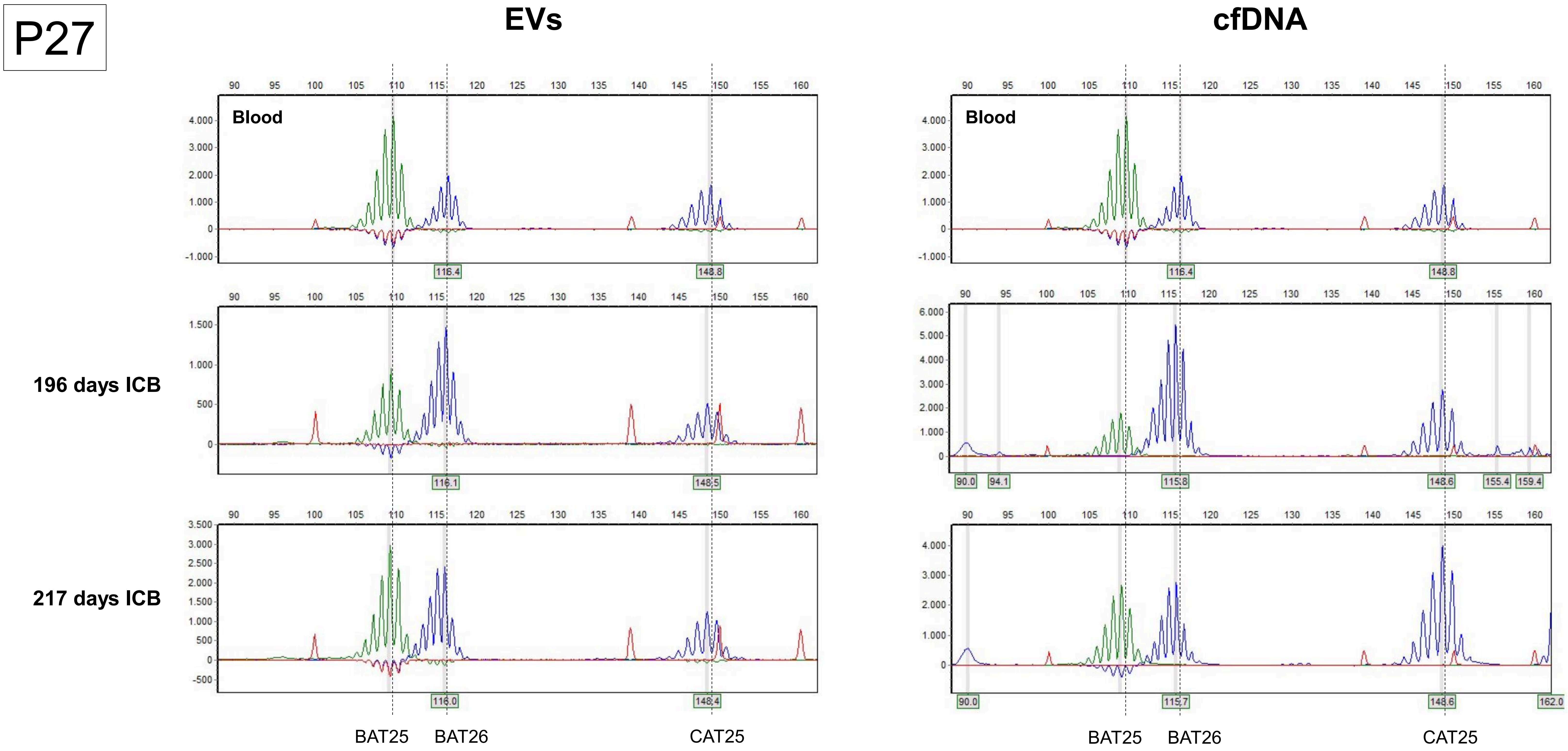
P27
EVs
cfDNA
Blood
Blood
196 days ICB
217 days ICB
116.4
148.8
116.4
148.8
116.1
148.5
90.0
94.1
115.8
148.6
155.4
159.4
116.0
148.4
90.0
115.7
148.6
162.0
BAT25
BAT26
CAT25
BAT25
BAT26
CAT25

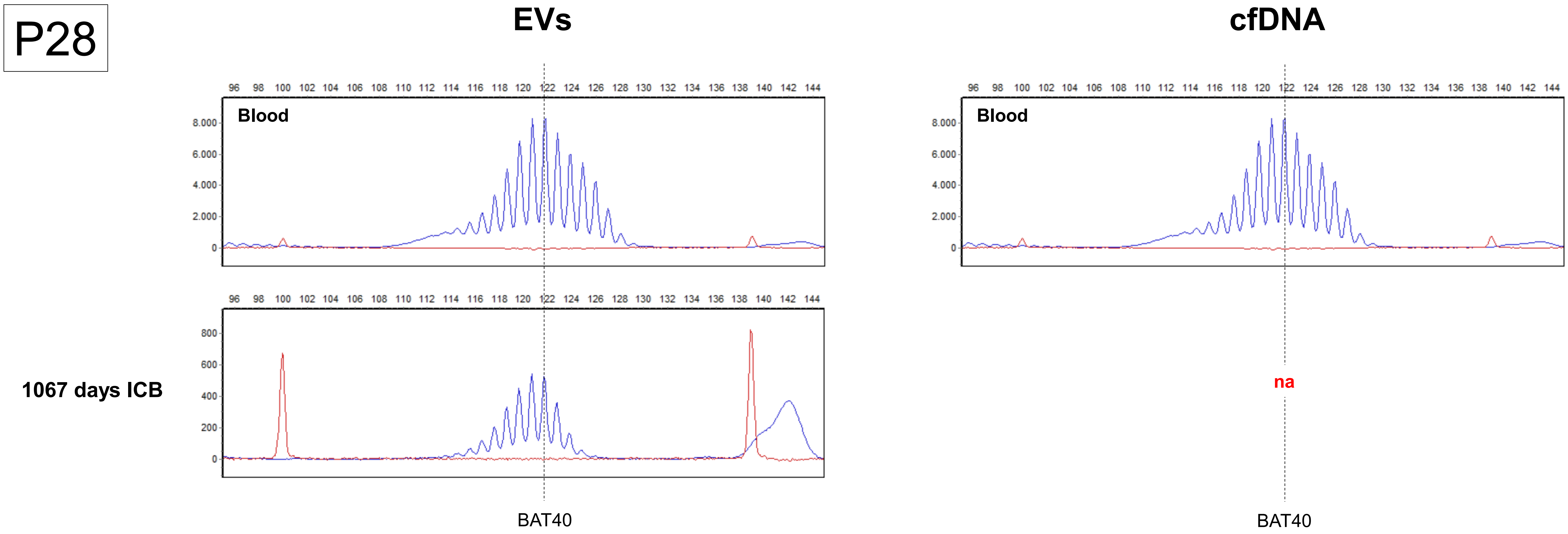

P28
EVs
cfDNA
Blood
Blood
1067 days ICB
na
BAT40
BAT40

P28

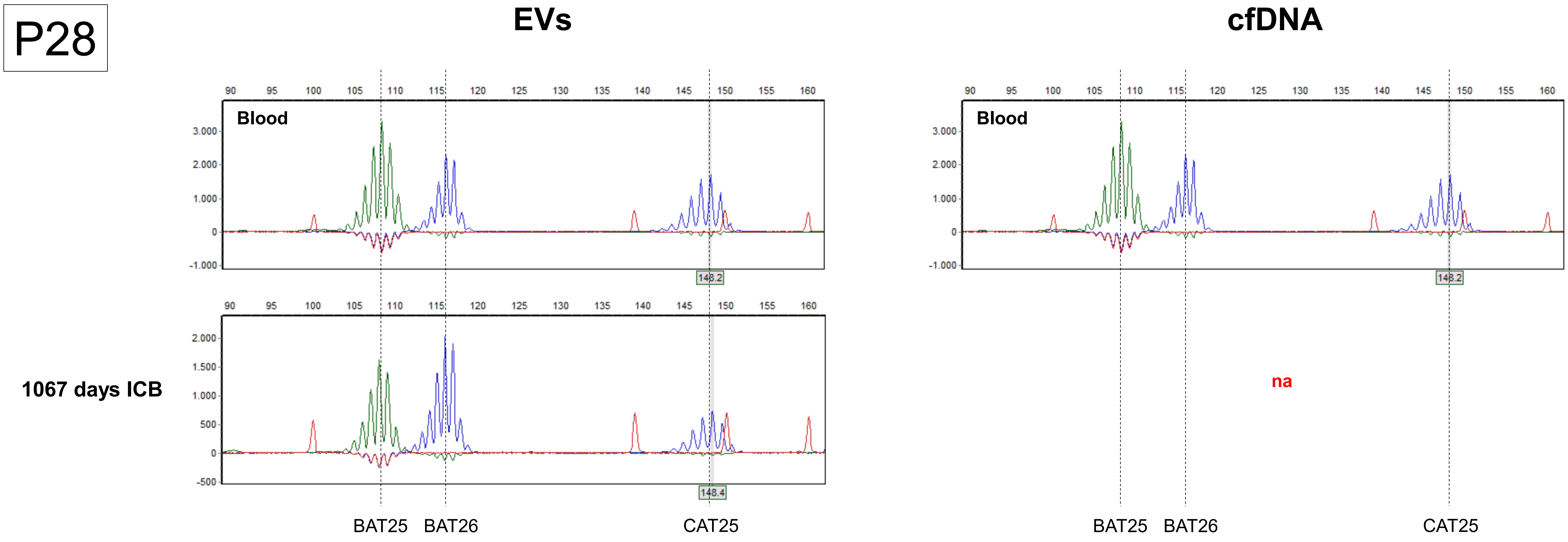

EVs
cfDNA
Blood
Blood
1067 days ICB
na
BAT25
BAT26
CAT25
BAT25
BAT26
CAT25
148.2
148.4
148.2
