# Supplementary material for: Detection of MSI signals from peripheral blood for monitoring response to immune checkpoint blockade therapy in patients with advanced microsatellite‐unstable gastrointestinal cancers: A pilot study
Source: Int J Cancer. 2026 Feb 16;158(12):3312–23. doi: 10.1002/ijc.70387 (PMC13106927; doi:10.1002/ijc.70387)
Supplement: Supplementary file 2 — Data S2. Supporting Information. [file IJC-158-3312-s002.pdf]

EXTENDED DATA 2

Tabular overview of the MSI analysis results from the EV DNA and cfDNA samples presented by individual markers (BAT40, BAT25, BAT26, CAT25) and overall MSI classification (MSI status).

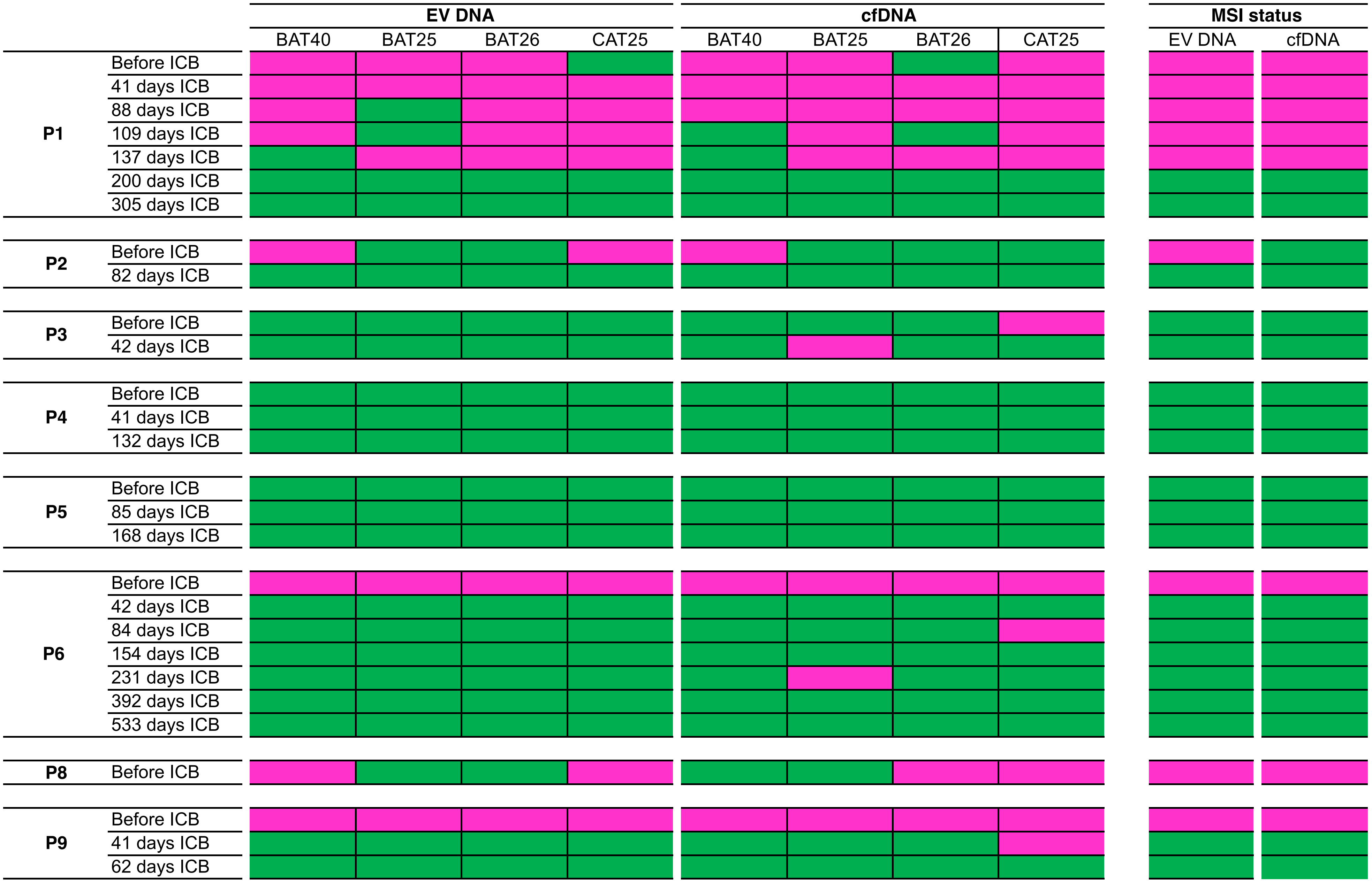
EV DNA
cfDNA
MSI status
BAT40
BAT25
BAT26
CAT25
BAT40
BAT25
BAT26
CAT25
EV DNA
cfDNA
P1
Before ICB
41 days ICB
88 days ICB
109 days ICB
137 days ICB
200 days ICB
305 days ICB
P2
Before ICB
82 days ICB
P3
Before ICB
42 days ICB
P4
Before ICB
41 days ICB
132 days ICB
P5
Before ICB
85 days ICB
168 days ICB
P6
Before ICB
42 days ICB
84 days ICB
154 days ICB
231 days ICB
392 days ICB
533 days ICB
P8
Before ICB
P9
Before ICB
41 days ICB
62 days ICB

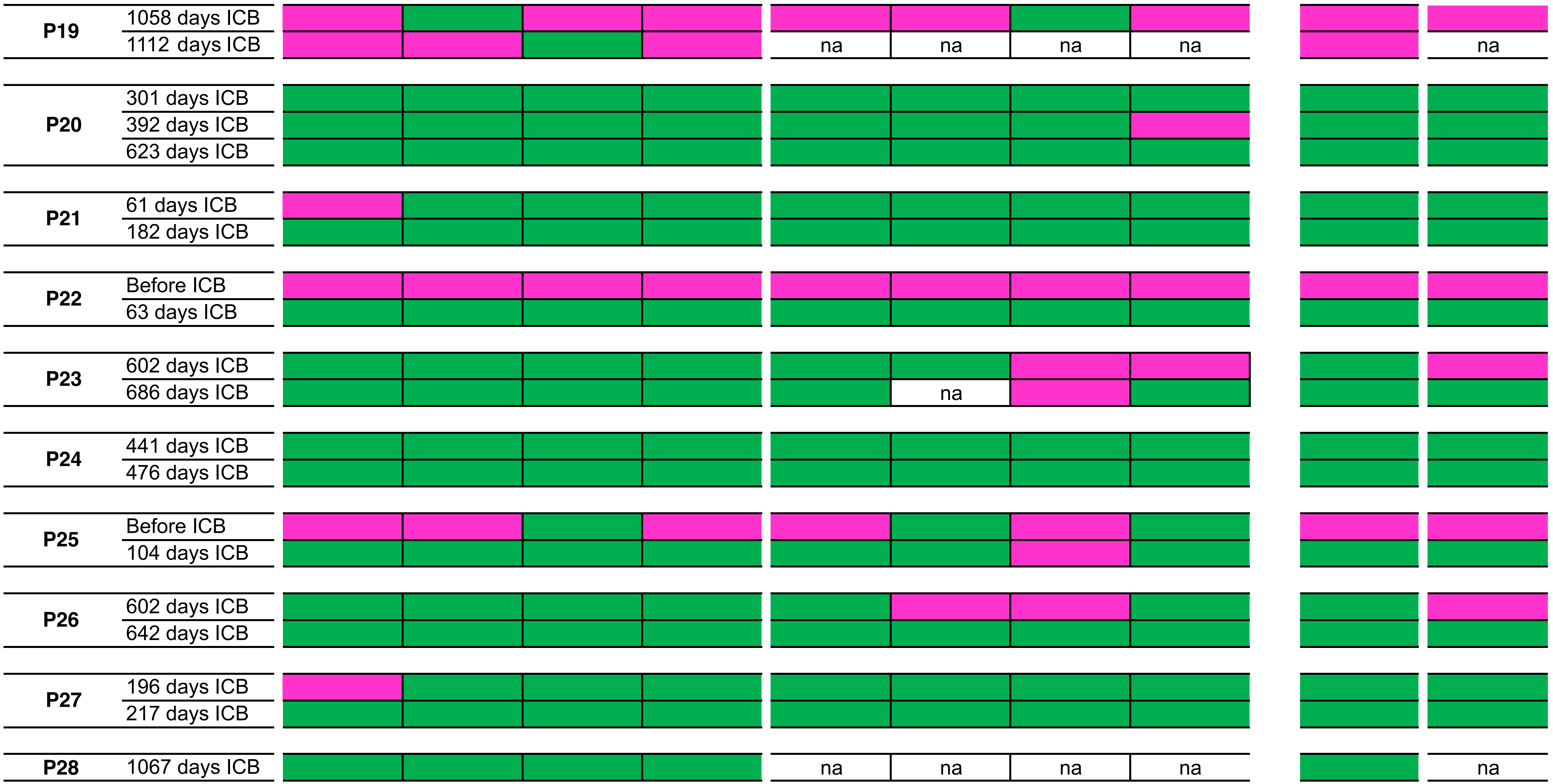
P19
1058 days ICB
1112 days ICB
na
na
na
na
na
P20
301 days ICB
392 days ICB
623 days ICB
P21
61 days ICB
182 days ICB
P22
Before ICB
63 days ICB
P23
602 days ICB
686 days ICB
na
P24
441 days ICB
476 days ICB
P25
Before ICB
104 days ICB
P26
602 days ICB
642 days ICB
P27
196 days ICB
217 days ICB
P28
1067 days ICB
na
na
na
na
na
