# Supplementary material for: Detection of MSI signals from peripheral blood for monitoring response to immune checkpoint blockade therapy in patients with advanced microsatellite‐unstable gastrointestinal cancers: A pilot study
Source: Int J Cancer. 2026 Feb 16;158(12):3312–23. doi: 10.1002/ijc.70387 (PMC13106927; doi:10.1002/ijc.70387)
Supplement: Supplementary file 3 — Data S3. Supporting Information. [file IJC-158-3312-s004.pdf]

EXTENDED DATA 3

MSI analysis of tumor samples.

*Asterisks mark patients from which “before therapy” sample for EV analyses was available. Blue triangles indicate peaks also observed in EV DNA (see Extended Data 1).

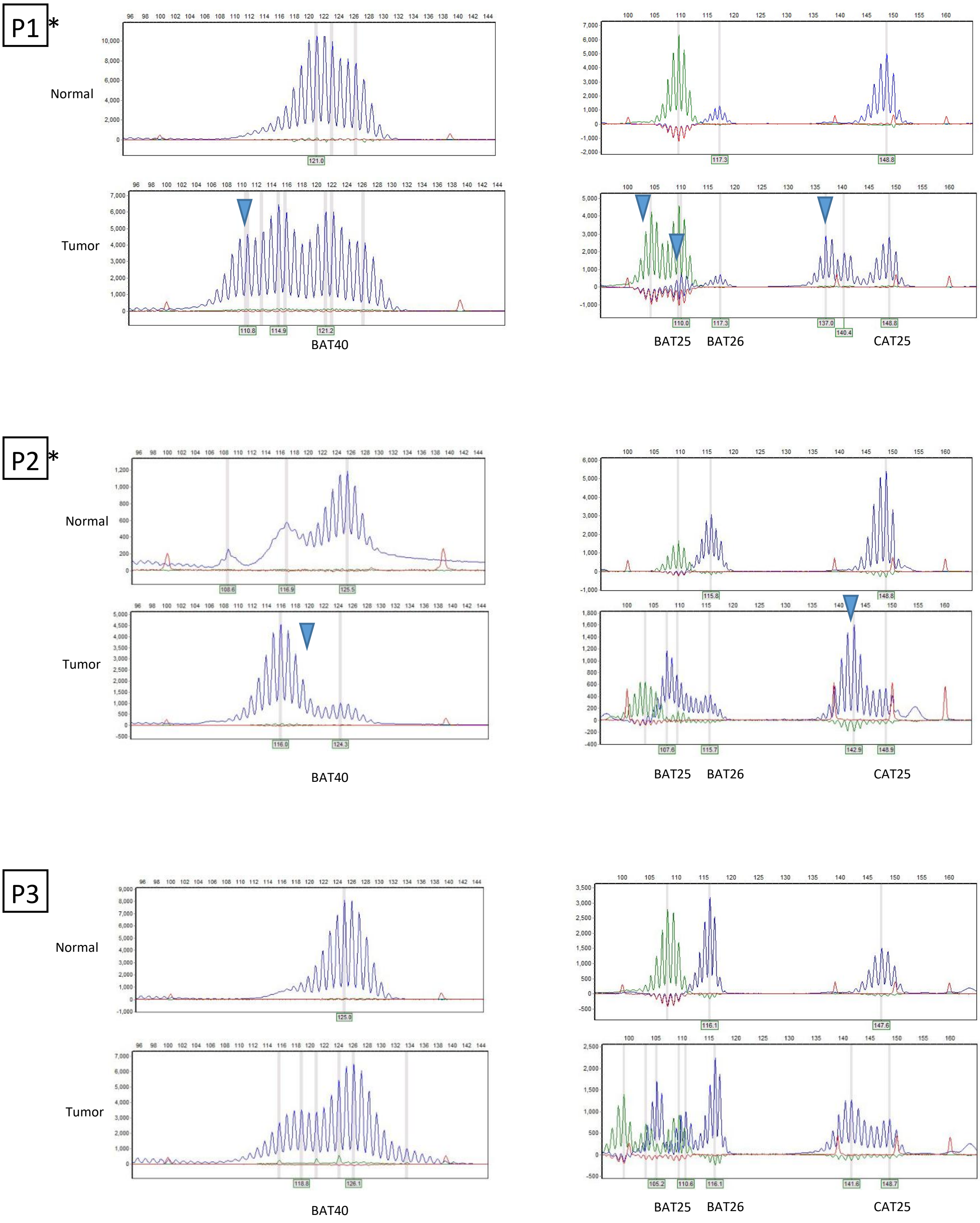
P1 *
Normal
Tumor
BAT40
BAT25
BAT26
CAT25
P2 *
Normal
Tumor
BAT40
BAT25
BAT26
CAT25
P3
Normal
Tumor
BAT40
BAT25
BAT26
CAT25

P4

Normal

Tumor

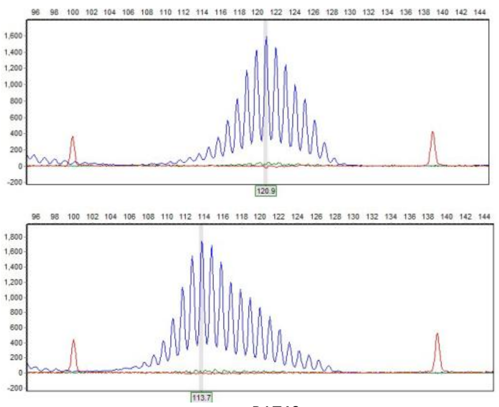

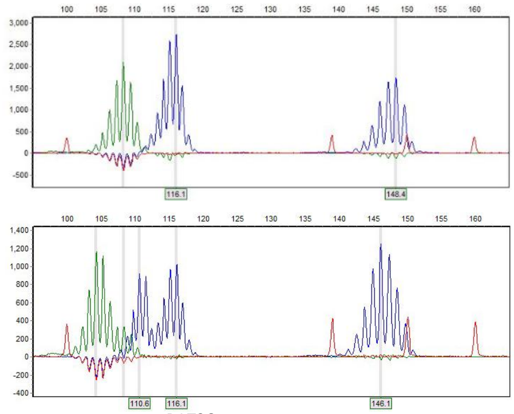

BAT40 BAT25 BAT26 CAT25

P5

Normal

External MSI analysis, no tumor tissue block available

Tumor

BAT40 BAT25 BAT26 CAT25

P6 *

Normal

External MSI analysis, no tumor tissue block available

Tumor

BAT40 BAT25 BAT26 CAT25

P8 *

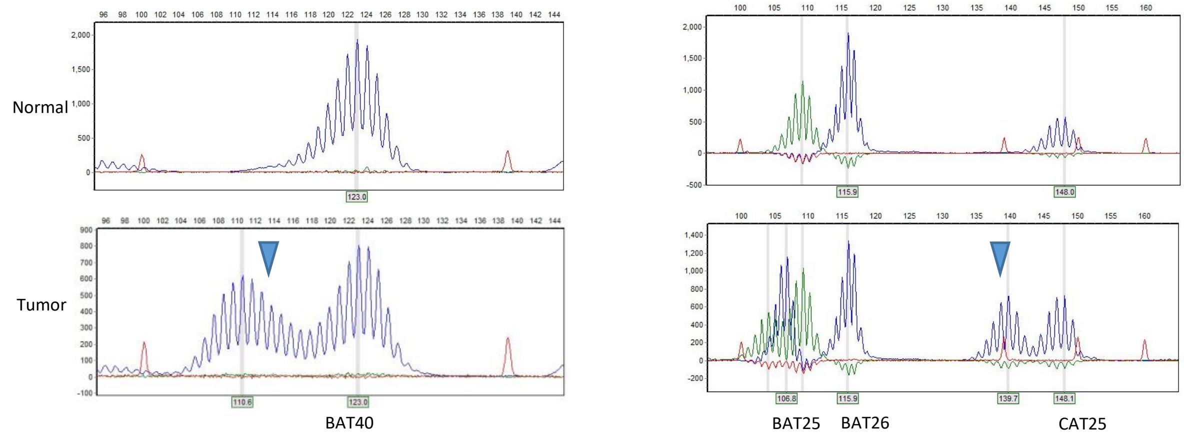
Normal
Tumor
BAT40
BAT25
BAT26
CAT25

P9 *

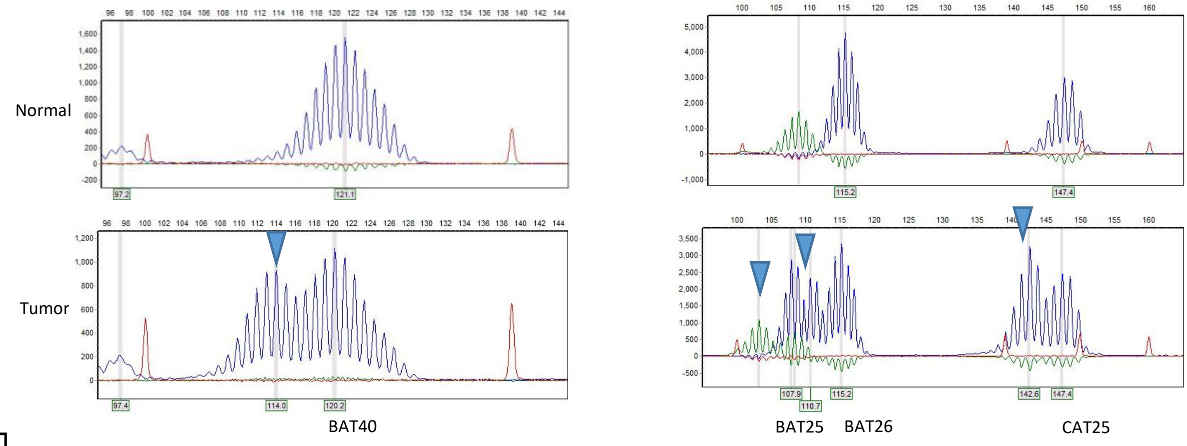
Normal
Tumor
BAT40
BAT25
BAT26
CAT25

P19

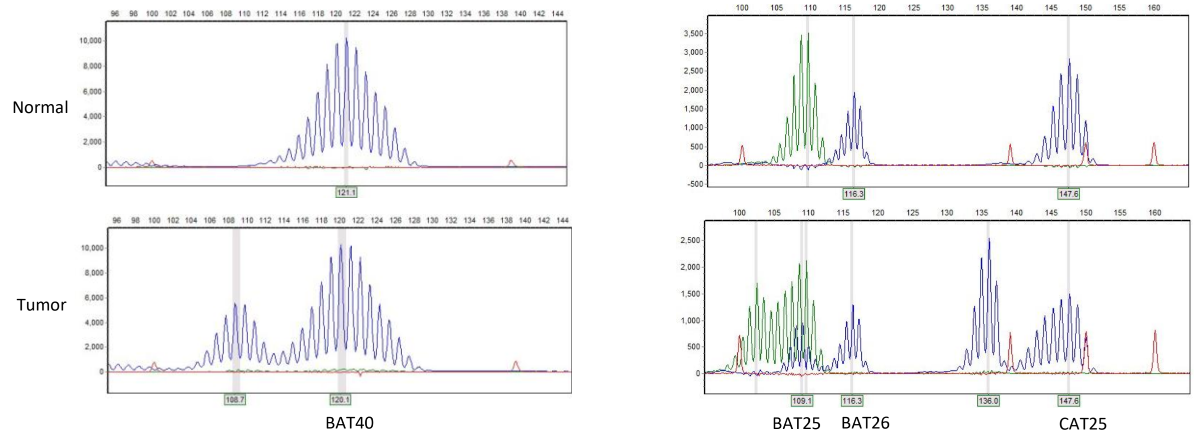
Normal
Tumor
BAT40
BAT25
BAT26
CAT25

P20

Normal

Tumor

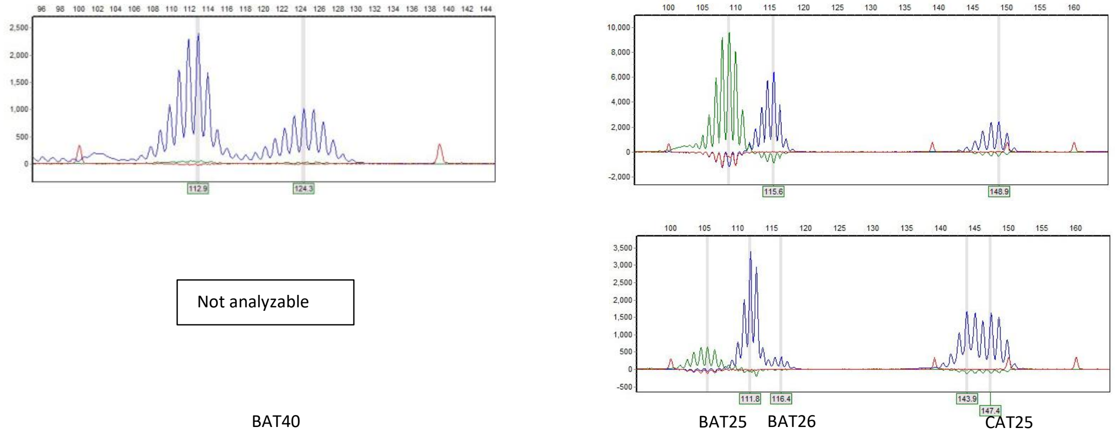

BAT40 BAT25 BAT26 CAT25

P21

Normal

Tumor

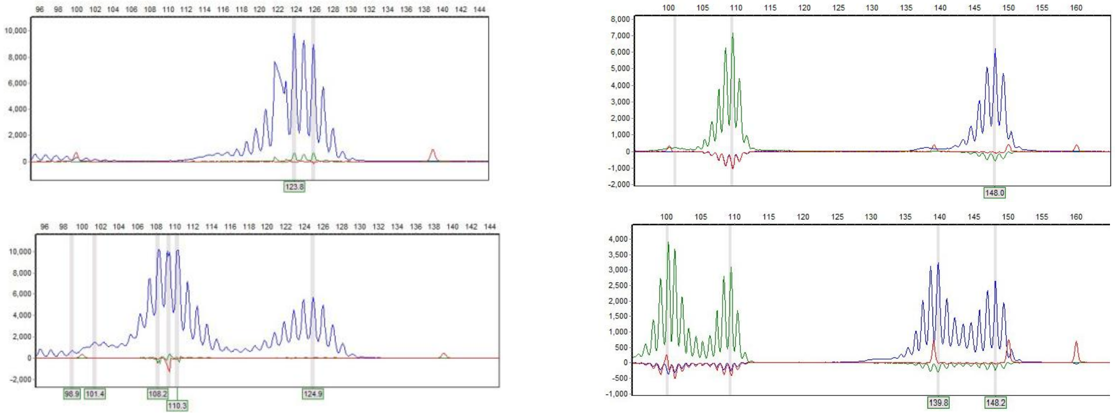

BAT40 BAT25 BAT26 CAT25

P22 *

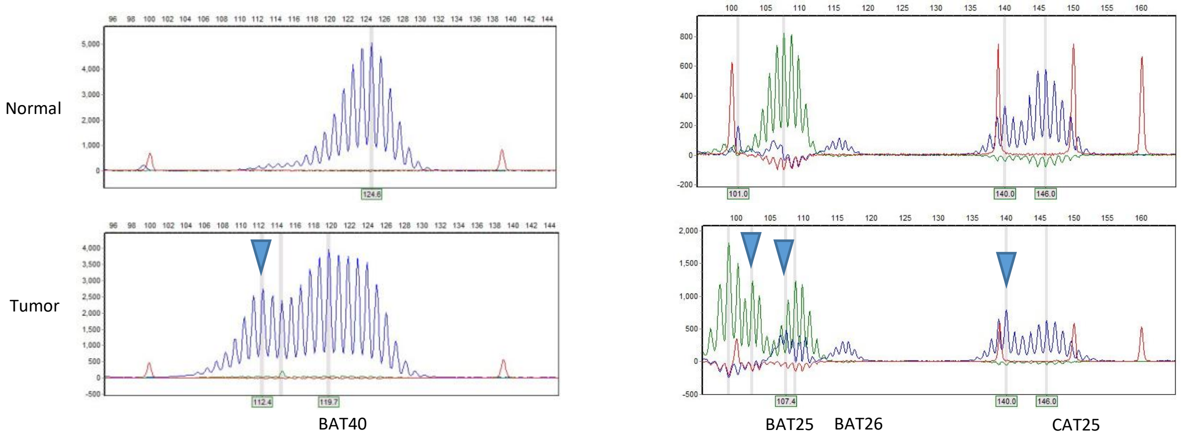
Normal
Tumor
BAT40
BAT25
BAT26
CAT25

P23

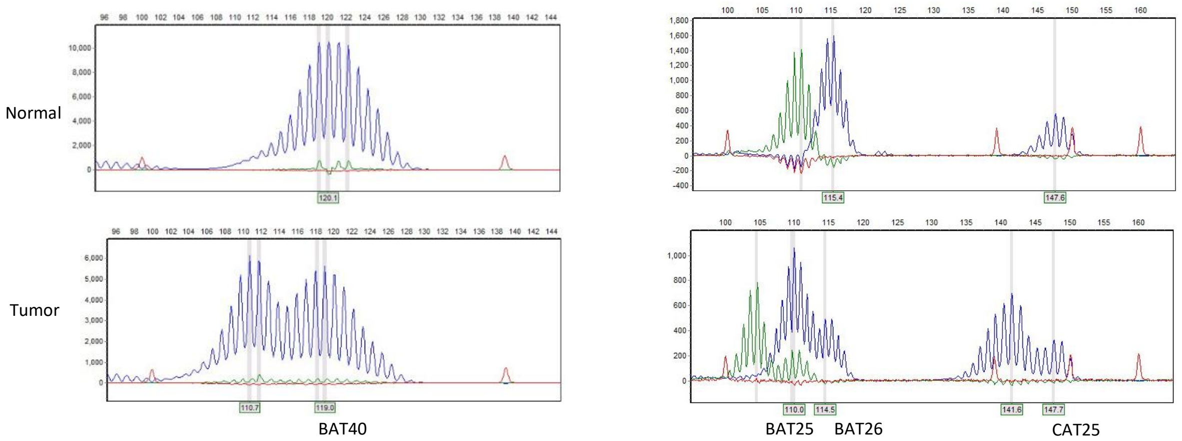
Normal
Tumor
BAT40
BAT25
BAT26
CAT25

P24

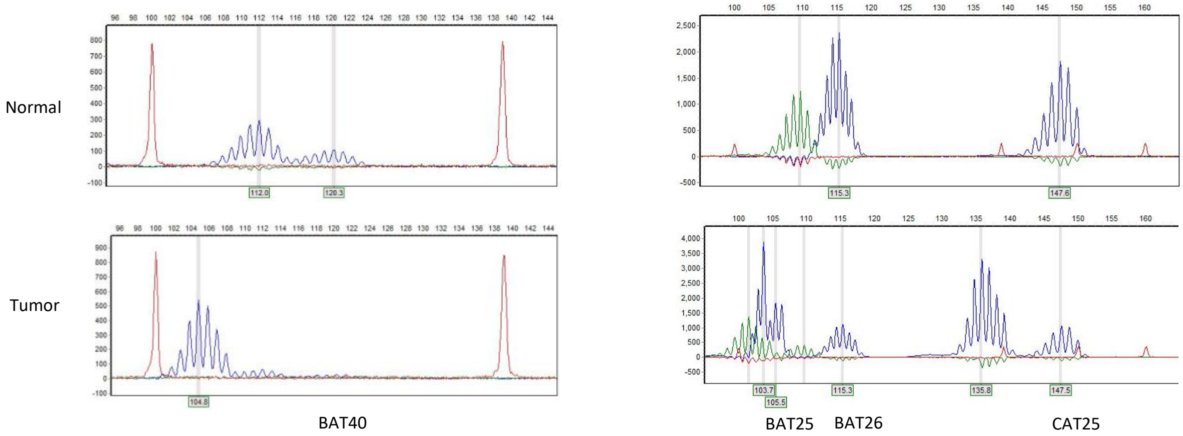
Normal
Tumor
BAT40
BAT25
BAT26
CAT25

P25 *

Normal

BAT40	BAT25 / BAT26 / CAT25
Not available	Not available

Tumor

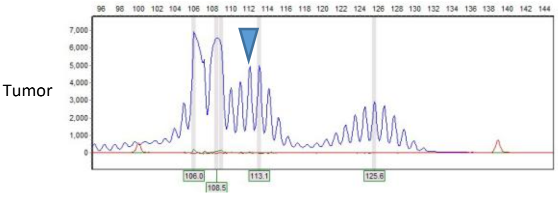

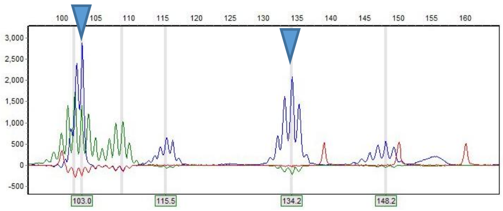

BAT40

BAT25 BAT26 CAT25

P26

Normal

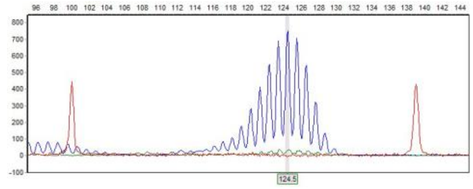

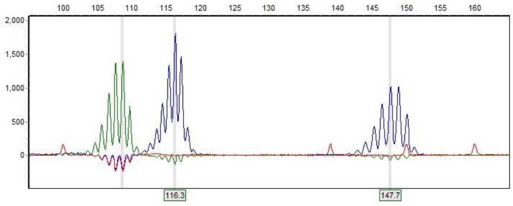

Tumor

Not analyzable

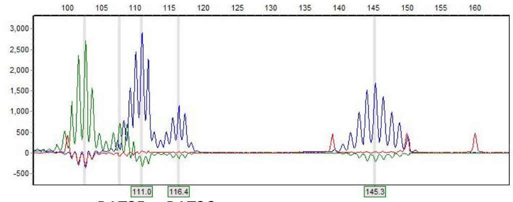

BAT40

BAT25 BAT26 CAT25

P27

Normal

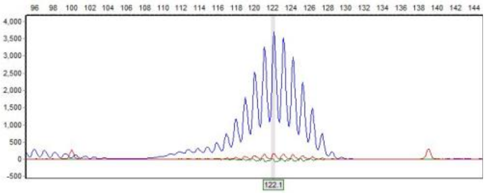

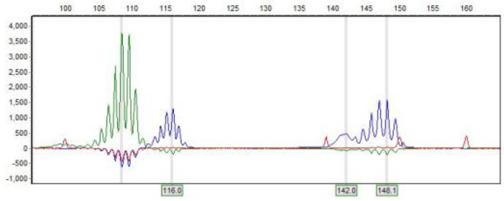

Tumor

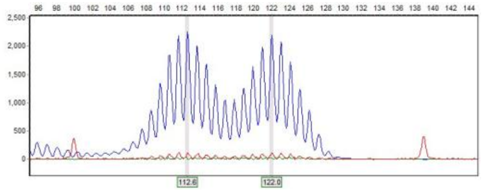

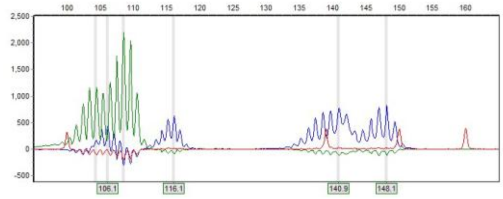

BAT40

BAT25 BAT26 CAT25

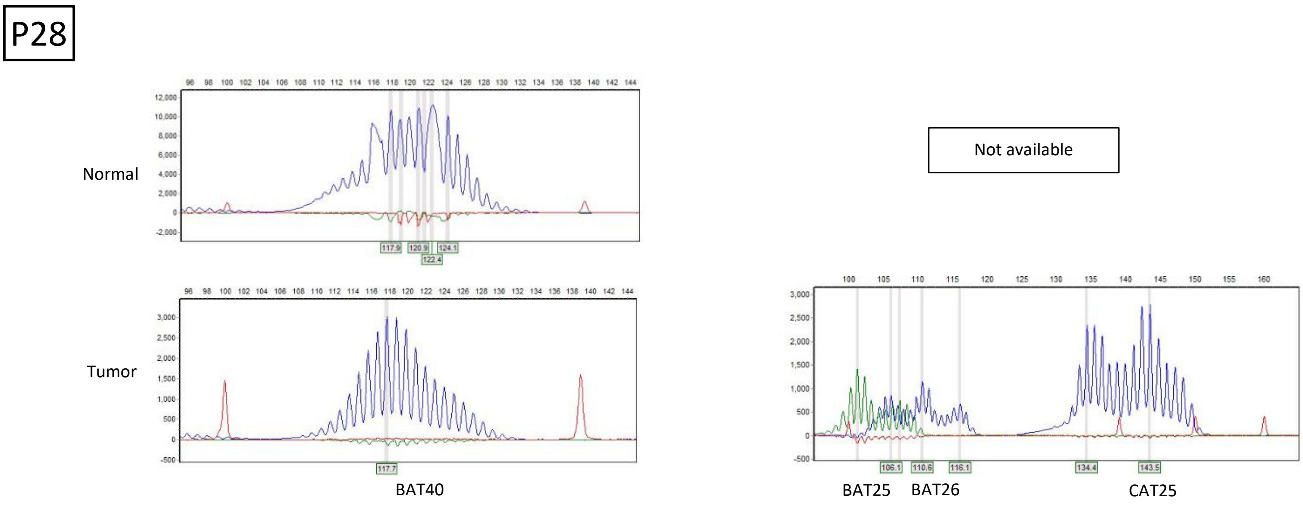

P28
Normal
Tumor
Not available
BAT40
BAT25
BAT26
CAT25
