# Supplementary material for: Detection of MSI signals from peripheral blood for monitoring response to immune checkpoint blockade therapy in patients with advanced microsatellite‐unstable gastrointestinal cancers: A pilot study
Source: Int J Cancer. 2026 Feb 16;158(12):3312–23. doi: 10.1002/ijc.70387 (PMC13106927; doi:10.1002/ijc.70387)
Supplement: Supplementary file 4 — Data S4. Supporting Information. [file IJC-158-3312-s001.pdf]

Supplementary Material

Detection of MSI signals from peripheral blood for monitoring response to immune checkpoint blockade therapy in patients with advanced microsatellite-unstable gastrointestinal cancers: a pilot study

Aysel Ahadova*, Lena Bohaumilitzky*, Thomas Walle, Joscha A. Kraske, Mirjam Tariverdian, Ulrike Ganserer-Schmitt, Ingrid Hausser-Siller, Vera Fuchs, Nina Nelius, Gizem Mehtap Erisen, Johannes Gebert, Albrecht Stenzinger, Dirk Jäger, Magnus von Knebel Doeberitz, Georg Martin Haag, Elena Busch*, Matthias Kloor*

*equal contribution

Table of contents

Supplementary Tables 2

Supplementary Figures 3

Supplementary Tables

Supplementary Table 1: Description of tumor load. Low = local recurrence, few small metastatic lymph nodes; Intermediate = big metastatic lymph nodes or other tumor mass; High = extensive metastasis with bulk and/or multiple liver metastasis.

Patient ID	Initial tumor load	Specification tumor load
P1	low	local recurrence, per continuitatem into os sacrum
P2	intermediate	multiple abdominal lymph nodes and peritoneal nodes
P3	low	abdominal lymph nodes, adrenal lesion
P4	low	soft tissue thoracic wall, singular osteolytic lesion
P5	high	multiple peritoneal tumor bulks up to 88 mm
P6	low	multiple small retroperitoneal lymph nodes
P7	intermediate	multiple adnominal lymph nodes
P8	low	local recurrence, pleural nodule
P9	high	multiple liver metastases up to > 40 mm
P19	intermediate	pelvic tumor lesion 32 mm
P20	high	multiple liver metastases up to > 40 mm
P21	high	multiple liver metastases
P22	high	multiple liver metastases up to 60 mm and soft tissue metastases
P23	intermediate	mesenteric lymph node conglomerate up to 57 mm
P24	intermediate	multiple abdominal lymph nodes, adrenal metastases
P25	low	local recurrence, peritoneal nodes
P26	high	multiple liver and lung metastases, lymph node metastases
P27	low	local recurrence
P28	high	multiple liver metastases, abdominal lymph nodes and peritoneal nodes

Supplementary Figures

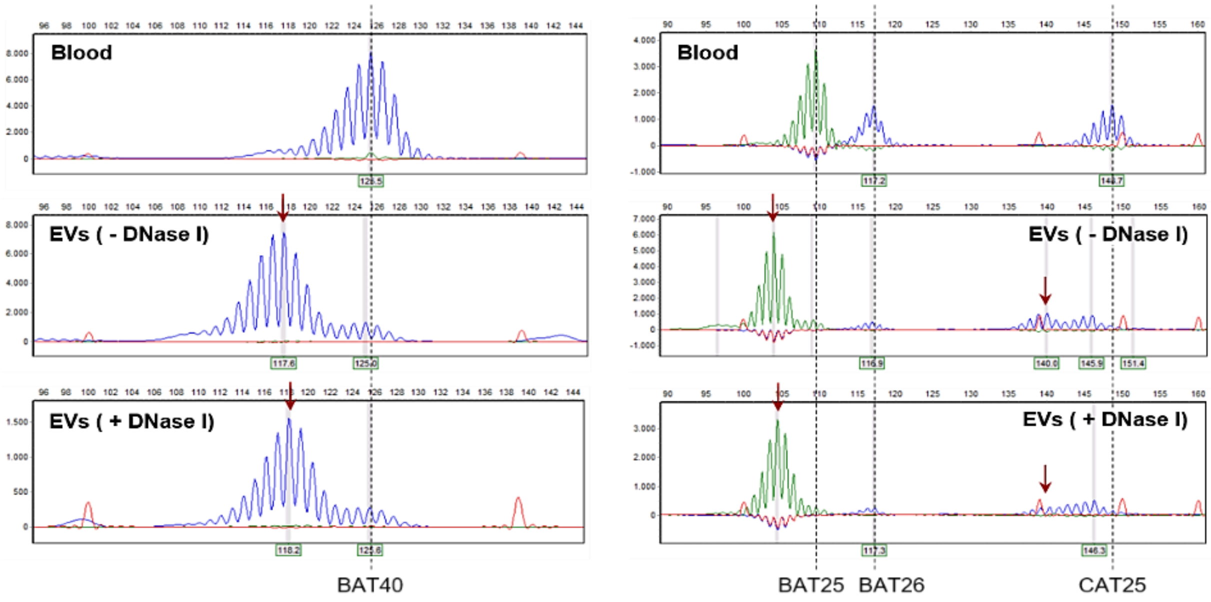

Supplementary Figure 1: MSI analysis of plasma-derived EV DNA after DNase I digestion of EV surface-associated DNA. DNA from whole blood was used as normal tissue control and is provided as reference. The markers BAT25, BAT26 and CAT25 were analyzed in a multiplex PCR, BAT40 was assessed individually. The amplicon length in bp is indicated on the top of each figure, the fluorescent signal intensity is provided on the left. Novel peaks indicative of MSI are labelled with red arrows.
